# Reducing health inequalities in disasters: A cross-sectional study of the viability of ‘vulnerability’ terminology and of priority lists in the UK

**DOI:** 10.1016/j.puhip.2024.100564

**Published:** 2024-12-20

**Authors:** Poppy Ellis Logan, Gabriella Rundblad, Marian Brooke Rogers, Richard Amlôt, Gideon James Rubin

**Affiliations:** a National Institute for Health and Care Research Health Protection Research Unit (NIHR HPRU) in Emergency Preparedness and Response, King's College London, United Kingdom; b Social Science & Public Policy: School of Education, Communication and Society, King's College London, United Kingdom; c Behavioural Science and Insights Unit, UK Health Security Agency, United Kingdom

**Keywords:** ‘Health inequalities’, ‘Health disparities’, ‘Health outcomes’, ‘Health equity’, ‘Disability’, ‘Disasters’, Emergency Preparedness

## Abstract

**Background:**

In disasters, people with certain characteristics repeatedly experience health inequalities. In the UK, people predicted to experience poorer health outcomes are often described as ‘vulnerable’. Various services compile lists of ‘vulnerable’ people eligible for interventions in disasters to reduce health disparities.

**Study aim:**

To explore the viability of current approaches to reducing health inequalities in disasters, we tested whether people typically described as ‘vulnerable’ by public health and emergency planners self-identify as 'vulnerable' in a disaster, and whether they are registered on a ‘vulnerability list’.

**Study design:**

We collected data from 5148 UK-based adults using a cross-sectional online survey from July–September 2022, using nationally representative quotas for age, gender, disability, and social grade.

**Methods:**

We calculated the proportions of respondents with perceived indicators of ‘vulnerability’ who self-described as 'vulnerable during a disaster’, and who reported being on a Priority Service Register or another ‘vulnerability list’. We used odds ratios to assess whether access to resources or risk mitigation plans explained low rates of self-identification as 'vulnerable' and registration.

**Results:**

Among people with perceived indicators of 'vulnerability', self-description as ‘vulnerable in a disaster’ ranged from 22.4 % (of people dependent on false teeth) to 60.7 % (of people reporting significant difficulty running errands alone). Registration on a Priority Service Register ranged from 11.4 % (of people who were pregnant) to 35.7 % (of people reporting difficulties dressing, bathing, or using the toilet independently). Respondents without alternative plans or resources were generally no more likely to consider themselves ‘vulnerable’ or be registered on a 'vulnerability list' than those with alternative plans or resources.

**Conclusions:**

Communications using the term 'vulnerable' may not reach target audiences. Using priority lists to reduce health disparities is impractical as most people facing inequitable risk are not registered. We suggest shifting UK terminology and discourse surrounding disaster risk, focussing on making mainstream strategies inclusive and accessible to reduce health inequalities in disasters.

## What this study adds

• Across a large sample of UK adults, most people facing disparate risk as indicated by unmet health requirements, who would be predicted to experience health inequalities in disasters, did not describe themselves as ‘vulnerable’ and were not signed up to a Priority Services Register or another ‘vulnerability list’.
• This was not because people had made their own arrangements to prepare for a disaster – people without backup plans or resources in place were generally no more likely to consider themselves ‘vulnerable’ or to be on a register than those with plans or resources.

## 1 Introduction

In the UK, the term 'vulnerable' is used to describe people with certain characteristics who are predicted to have significantly poorer health outcomes than the wider population during and after hazardous events [1,2]. To reduce these health disparities during disasters, various UK services collate lists of ‘vulnerable’ people identified against these characteristics [3]. This builds on an idea that if services share the details compiled, local authorities can provide aid to ‘vulnerable’ people in an emergency [3,4]. The Priority Service Register (PSR) system comprises opt-in lists of ‘vulnerable’ people compiled by utilities companies [1]. The PSRs hypothetically equip utilities companies to protect ‘vulnerable’ people during disruption to essential services [1,3].

Reliance on ‘vulnerability lists’ is increasingly criticised. During the COVID-19 pandemic, the UK Government used various datasets to identify those required to ‘shield’ via a 12-week home isolation period, intending to supply them with the resources and information they would require [5]. The lists were inaccurate, incomplete, and misinterpreted [5,6]. Of people eventually identified, only 23.2 % ever received the support offered by the scheme [5].

The sole provision, and consequent reliance on inexhaustive, medicalised lists in COVID-19 failed conceptually to embed the “social and human rights models” of disability set out by the UK Government's Equalities Office [6], and resulted in >300 legal claims of breaches to the Equality Act 2010 and the Human Rights Act 1998 [[9], [10], [11]].

The poor performance of the COVID-19 Clinically Extremely Vulnerable (CEV) list may relate to its use of the word ‘vulnerable’. The concept of ‘vulnerability’ in disasters, and the use of the term ‘vulnerable’ in public health are contested [7,8] and disliked by many people who prefer to be described in different terms. ‘Vulnerability lists’ that do not use this term, such as PSRs, may be more popular and effective.

Our study assessed whether using the term ‘vulnerable’ and whether strategically relying on ‘vulnerability lists’ such as PSRs can be justified, by examining whether self-description of ‘vulnerability’ in disasters aligns with the term's intended meaning, and assessing rates of registration on current ‘vulnerability lists’.

Specifically, we investigated:

1) Whether people with perceived indicators of vulnerability consider themselves to be ‘vulnerable’ in a disaster;
2) What proportion of people with perceived indicators of vulnerability are registered as a ‘vulnerable’ person on any list held by agencies including: their GP surgery or pharmacy, a local council or local authority, social care services, or any operators of essential services (electricity, gas, water, or telecommunications);
3) What proportion of people with perceived indicators of vulnerability have access to resources that mitigate against disparate disaster risk, independent of relying on external aid via a ‘vulnerability list’, and;
4) What proportion of people with or without access to such resources consider themselves ‘vulnerable’ and are on a Priority Services Register?

## 2 Methods

### 2.1 Design

We used a cross-sectional online survey designed to assess nationwide preparedness for, resilience to, and communication preferences in relation to disasters (the Measure of All-hazard Preparedness (MAP)). The MAP reported on a sample of 5148 participants, aged 18 or over and living in the UK.

### 2.2 Participants

Participants were recruited from an existing panel maintained by a market research company (Savanta). Panel members had previously actively opted-in to receiving invitations to diverse market research surveys, receiving payment for each completed survey. The MAP sample aimed to be nationally representative, using quotas for age, gender and NUTS1 UK region according to the latest Office for National Statistics mid-year population estimates [[12], [13], [14]]; disability according to estimates from the Family Resources Survey [15]; and social grade according to the 2011 Census [14].

### 2.3 Setting

Data collection occurred online from July 11, 2022–September 12, 2022.

### 2.4 Measures

The MAP contains many items, with only a subset analysed for this study. The full questionnaire is available at https://doi.org/10.17605/OSF.IO/KQUHX.

To assess self-perception of vulnerability, participants were asked ‘would you consider yourself to be vulnerable during a disaster?’ Possible responses were ‘yes’, ‘no’, ‘maybe’, ‘prefer not to say’, and ‘don't know’.

We assessed registration on any ‘vulnerability list’ by asking ‘are you or anyone in your home registered as a ‘vulnerable’ person with any of the following: your GP surgery or pharmacy; your local council or local authority; social care services; your electricity, gas, water or telephone company’. Response options were ‘yes - I am registered’, ‘yes - someone I care for is registered’, ‘I might be registered, but I'm not sure’, ‘someone I care for might be registered, but I'm not sure’, ‘no’, and ‘prefer not to say’. Participants could select all that applied to them.

If the respondent answered ‘yes – I am registered’, and/or ‘yes - someone I care for is registered’ to the previous question, they were subsequently asked ‘are you or anyone you care for in your home registered as a ‘vulnerable’ person with any of the following:’ with the response options being ‘your GP surgery or pharmacy’, ‘your local council or local authority’, ‘social care services’, ‘your electricity, gas, water, or telephone company’, ‘somewhere else (please specify)’ and ‘prefer not to say’. These response options were presented in a randomised order and participants were asked to select all that applied.

We asked about a range of perceived indicators of vulnerability, split into questions asking firstly about characteristics, conditions, and difficulties doing things independently, and secondly about whether the respondent's health would be affected by losing access to various resources. Examples of the former set of questions included asking participants whether they would refer to themselves as disabled, whether they were pregnant, whether they would describe themselves as either d/Deaf or likely to experience significant difficulty hearing, whether they were blind or had significant difficulty seeing, and whether they had a condition which can significantly affect their speech (see Table 1, Table 2 for full list).

Questions asking whether people's health would be affected by losing access or experiencing interruption to various resources or services were asked together in a randomised carousel, and are reported in Table 2. For example, we asked: “During an emergency, would your health or the health of anyone you care for (including infants) be significantly affected if you or they could not access or use any of the following:”, and for each resource or service listed, the participant selected either: ‘Yes-my health would be affected’, ‘Yes – the health of someone I care for would be affected’, ‘Yes – both my health and the health of someone I care for would be affected’, ‘No – neither my health nor the health of someone I care for would be affected’, or ‘Prefer not to say’.

To assess whether people would be able to meet resource-dependent health requirements in a disaster, we also asked about access to backup supplies or alternative plans to mitigate against disparate risk (listed in Table 3). These questions were only asked to participants who had previously indicated having a relevant condition, characteristic or requirement.

### 2.5 Public involvement

Several rounds of public involvement were completed during the development of the survey, bringing together diverse members of the public and experts into shared (virtual) spaces to discuss and collaborate on question items and survey design over several months, with multiple waves of review and feedback integration. Written input was received from the Maudsley Biomedical Research Centre's Feasibility and Acceptability Support Team for Researchers. Written feedback was also received from a panel of people with conditions, characteristics and disabilities with and without lived experiences of disasters. An online group discussion was also completed with this group. Changes made based on the feedback included the provision of an estimate for how long the survey would take, an explanation that it should be completed in one session, and additional answer options for items relating to difficulties seeing or hearing.

Written feedback was also received from members of the British Academy of Social Workers, and meetings were held with frontline responders to discuss the items required for UK disaster preparedness. Feedback led to the inclusion of questions about certain backup plans, such as written copies of medications and dosages.

The UKHSA Behavioural Science Unit also advised on data points that the UKHSA would find particularly helpful, such as questions about reliance on electronic medical equipment. Following this, people who rely on electronic home medical equipment and certain feeding supplies were contacted additionally for their input over the wording of questions on these resources.

### 2.6 Analysis

We assessed rates of PSR use by calculating the proportion of participants who reported perceived indicators of vulnerability and who selected ‘Your electricity, gas, water, or telephone company’ in response to the question asking which ‘registers’ their household was on. We also calculated rates of participants who were personally registered on any of the ‘vulnerability lists’ provided in the answer options.

We used cross-tabulations calculated in SPSS to assess:

• the proportion of respondents per condition, characteristic (Table 1) or resource dependency (Table 2) who: self-described as ‘vulnerable’ during a disaster; were on any ‘vulnerability list’; or said anyone in their household was on a PSR;
• the proportion of respondents per perceived marker of vulnerability who reported having or not having access to relevant resources (Table 3);
• the association (using odds ratios and 95 % confidence intervals) between having backup resources, and either self-describing as ‘vulnerable’ in a disaster or reporting that your household was on a PSR (Table 4).

## 3 Results

A total of 3313 participants reported one or more perceived indicator of vulnerability. Of these, the proportion who considered themselves ‘vulnerable’ during a disaster ranged between 22.4 % (those with false teeth) to 60.7 % (for participants with significant difficulty running errands such as visiting the pharmacy or shopping alone). Only 57.7 % of people who would refer to themselves as ‘disabled’ considered themselves ‘vulnerable’ in a disaster. Table 1, Table 2 provide the frequencies and percentages of these outcomes across each condition, characteristic and requirement.

**Table 1:** Proportion of respondents by characteristic or condition who: consider themselves ‘vulnerable’ during a disaster; are personally on any ‘vulnerability list’, or said their household was on a PSR.

	Considers self as ‘vulnerable’ (%/n)	Personally registered on any list (%/n)	Anyone in household on a PSR (%/n)
Would you refer to yourself as disabled?	57.7 % (480/832)	48.2 % (401/832)	35.8 % (298/832)
Are you pregnant?	24.8 % (26/105)	32.4 % (34/105)	11.4 % (12/105)
Would you describe yourself as either d/Deaf or likely to experience significant difficulty hearing?	40.2 % (70/174)	35.0 % (62/174)	25.3 % (44/174)
‘*Yes, but not when wearing hearing aids*’	38.6 % (86/223)	39.0 % (87/223)	32.7 % (73/223)
Do you have any other condition(s) that may stop you from hearing alarms or other noise alerts (such as a fire bell, sirens, or megaphone announcements)?	60.0 % (63/105)	51.4 % (54/105)	29.5 % (31/105)
Is sign language your preferred language?	35.5 % (27/76)	50.0 % (38/76)	30.2 % (23/76)
Do you have a condition which can significantly affect your speech?	55.7 % (73/131)	48.9 % (64/131)	25.2 % (33/131)
Are you blind or do you have significant difficulty seeing?	50.6 % (40/79)	44.3 % (35/79)	32.9 % (26/79)
‘*Yes, but not when wearing contact lenses or glasses*’	28.0 % (228/813)	23.7 % (193/813)	19.3 % (157/813)
Separate to a hearing or visual impairment, do you have a health condition that can make it hard to understand or talk to people at times?	46.0 % (165/359)	40.4 % (145/359)	24.5 % (88/359)
Do you have a condition or specific need (including for regular medication) that would be hard for you to explain to a doctor on your own without having it written down?	57.3 % (189/330)	52.1 % (172/330)	29.4 % (97/330)
Do you currently have a reduced or impaired sense of smell which could prevent you from being able to sense a gas leak, burning/smoke, or soiled clothing/nappies?	34.6 % (85/246)	32.5 % (80/246)	25.4 % (60/246)
Do you experience significant difficulty concentrating, remembering, or making decisions?	37.9 % (305/804)	35.1 % (282/804)	21.2 % (176/804)
In an emergency, would you have significant difficulty doing the following?			
1) Hearing, understanding, or following spoken instructions in English on your own	40.4 % (138/342)	44.4 % (152/342)	26.9 % (92/342)
2) Speaking in English to someone you don't know on your own	37.9 % (127/335)	41.8 % (140/335)	21.5 % (72/335)
3) Moving about your home without aid or assistance	51.9 % (255/491)	49.1 % (241/491)	33.4 % (164/491)
4) Dressing, bathing, or using the toilet on your own	52.2 % (199/381)	53.8 % (205/381)	35.7 % (136/381)
5) Feeding yourself on your own	43.3 % (143/330)	49.7 % (164/330)	28.8 % (95/330)
Because of a disability and/or health condition, would you have significant difficulty doing errands alone such as visiting a pharmacy or shopping?	60.7 % (390/643)	51.0 % (328/643)	35.3 % (227/643)

**Table 2:** Proportion of respondents with resource-dependent health requirements who: consider themselves ‘vulnerable’ during a disaster; are personally on any ‘vulnerability list’; or said their household was on a PSR.

Participants who stated that their health would be significantly affected if they could not access or use the following:	Considers self as ‘vulnerable’ (%/n)	Personally registered on any list (%/n)	Anyone in household on a PSR (%/n)
Resources for which alternative access or preparedness was not assessed^a^			
A caregiver	49 % (159/324)	50.0 % (162/324)	33.0 % (107/324)
A service animal	39.4 % (69/175)	45.7 % (80/175)	20.0 % (35/175)
Electrical devices that call for help if you fall, or have a problem at home (e.g., personal alarms, security systems including telecare)	42.6 % (139/326)	45.4 % (148/326)	27.0 % (88/326)
Electrically powered assistive technology (e.g., communication aids/AAC)	41.3 % (100/242)	46.3 % (112/242)	26.0 % (63/242)
Controlled medications/medications that are only prescribed for up to 30 days at a time^b^	33.8 % (452/1339)	34.3 % (459/1339)	24.9 % (333/1339)
Electric wheelchair/scooter	47.2 % (119/252)	54.0 % (136/252)	34.5 % (87/252)
Home medical equipment that does not rely on electricity (e.g., walkers, manual wheelchairs, specialist mattresses)	51.1 % (178/348)	52.3 % (182/348)	31.9 % (111/348)
Sanitary products for medical conditions (e.g., urinary supplies like catheters and ostomy supplies)	34.9 % (113/324)	42.3 % (137/324)	24.1 % (78/324)
Resources for which alternative access or preparedness **was** assessed^c^			
Prescribed foods, feed, or feeding supplies	36.5 % (112/307)	41.4 % (127/307)	21.5 % (66/307)
Other specific foods (e.g., due to sensory needs) that must be refrigerated or cooked	36.9 % (121/328)	41.8 % (137/328)	22.9 % (75/328)
Medically prescribed food supplements	36.7 % (110/300)	43.7 % (131/300)	21.7 % (65/300)
Refrigerated medications	41.1 % (161/392)	44.9 % (176/392)	26.3 % (103/392)
Other prescribed medications, including ointments and creams	31.5 % (408/1295)	31.5 % (408/1295)	24.6 % (319/1295)
Home medical equipment that requires electricity (e.g., oxygen pumps, dialysis machines, CPAP devices, hoists, lifts)	43.1 % (128/297)	48.8 % (145/297)	31.6 % (94/297)
Equipment to help you manage your body temperature that is needed due to a medical condition	39.6 % (97/245)	46.9 % (115/245)	25.3 % (62/245)
Incontinence supplies (e.g., wearable pads)	44.1 % (165/374)	44.7 % (167/374)	29.4 % (110/374)
Other medical supplies (e.g., syringes, blood sugar monitoring strips or oxygen cylinders)	44.6 % (179/401)	47.9 % (192/401)	28.2 % (113/401)

a We did not determine availability or plans to access these resources during a disaster.

b Access to supplies of Controlled Medications is partially reflected by responses to having two weeks' worth of ‘Prescribed Medications’. However, it is illegal to stockpile Controlled Medications so individuals cannot intentionally prepare two weeks-worth for a disaster, by definition.

c Only these items are carried into Table 3.

Registration on a PSR for the characteristics and conditions asked about (see Table 1) ranged from 11.4 % (for respondents who were pregnant) to 35.7 % (for respondents who would have had difficulty dressing, bathing or using the toilet on their own). Registration was also low for respondents whose health was resource-dependent (Table 2). For example, only 31.6 % of respondents reliant on home medical equipment that requires electricity (e.g., oxygen pumps, dialysis machines, CPAP devices, hoists, lifts) were on a PSR.

Depending on the requirement, between 19.1 % and 89.4 % of respondents were unaware of having any backup resources or plan in place (Table 3). Table 4 shows the proportion of people with and without access to backup plans or resources who consider themselves ‘vulnerable’ in a disaster, or whose household was on a PSR. Overall, those without resources were less likely to self-describe as ‘vulnerable’ than those with access to resources. Statistically significant differences in self-description of ‘vulnerability’ were only seen for people with copies of a list of their medication/dosages (OR 0.56, CI 0.46–0.68, p < 0.001), spare false teeth (OR 0.51, CI 0.36–0.73, p < 0.001), and incontinence supplies (OR 0.42, CI 0.26–0.67, p < 0.001).

**Table 3:** Proportion of respondents with resource-dependent health requirements who can meet each requirement in a disaster.

Resource	Access to resource		
	Yes	No	‘I don't know’
Characteristic, health condition or needs written down due to difficulties explaining to a doctor alone	60.6 % (200/330)	30.9 % (102/330)	8.5 % (28/330)
Copies of a written list of medication and dosage	52.3 % (1060/2028)	45.1 % (914/2028)	2.7 % (54/2028)
Equipment to keep refrigerated medication cold if evacuating (e.g. a coolbox)	52.7 % (188/357)	42.3 % (151/357)	5.0 % (18/357)
Extra glasses or contact lenses	66.3 % (536/808)	30.8 % (249/808)	2.8 % (23/808)
Spare fully charged hearing aid batteries^a^	77.3 % (231/299)	17.1 % (51/299)	5.7 % (17/299)
Spare false teeth/dentures	10.6 % (153/1450)	84.6 % (1227/1450)	4.8 % (70/1450)
Alternative power source for electric medical equipment (or refrigerated medication)	23.3 % (127/544)	68.8 % (374/544)	7.9 % (43/544)
Temperature management equipment that works in a power cut (e.g. heat pads, battery operated fans)	39.1 % (88/225)	53.8 % (121/225)	7.1 % (16/225)
Incontinence supplies	61.0 % (188/308)	31.8 % (98/308)	7.1 % (22/308)
Two weeks worth of the following:			
1) Prescribed foods, feed, or feeding supplies	55.1 % (125/227)	33.0 % (75/227)	11.9 % (27/227)
2) Other specific foods (e.g. due to sensory needs) that must be refrigerated or cooked	51.3 % (140/273)	41.8 % (114/273)	7.0 % (19/273)
3) Medically prescribed food supplements	53.2 % (126/237)	38.0 % (90/237)	8.9 % (21/237)
4) Other medical supplies (e.g. syringes, blood sugar monitoring strips or oxygen cylinders).	66.0 % (223/338)	28.1 % (95/338)	5.9 % (20/338)
5) Prescribed medications	80.9 % (1551/1918)	17.1 % (328/1918)	2.0 % (39/1918)

Where totals do match with totals in the previous tables for the corresponding needs, this is because those who responded ‘Prefer not to say’ or ‘This does not apply to me’ in the question on access to resources were not included. For example, 374 people were asked about incontinence supplies because they stated previously that they needed these, but 66 responded ‘this does not apply to me’ or ‘Prefer not to say’ in response to the question “Do you have incontinence supplies?”.

a Anybody who responded ‘yes’ to experiencing difficulty hearing (397) was asked about hearing aids, because many people experience significant difficulty hearing even with hearing aids.

**Table 4:** Proportion of respondents with/without access to backup supplies who consider themselves as ‘vulnerable’ in a disaster, and/or who are on any PSR.

Resource	% Consider themselves ‘vulnerable’ in a disaster				% Anyone in household on a PSR			
	Resource available (n)	Resource not known to be available (‘no’ or ‘not sure’) (n)	Odds ratio and 95 % CI (ref those with resource)	P-value	Resource available (n)	Resource not known to be available (‘no’ or ‘not sure’) (n)	Odds ratio and 95 % CI (ref those with resource)	P-value
Characteristic, health condition or needs written down due to difficulties explaining to a doctor alone	60.0 % (120/200)	53.1 % (69/130)	0.75 (0.48–1.18)	0.215	33.0 % (66/200)	23.8 % (31/130)	0.64 (0.39–1.05)	0.076
Copies of a written list of medication and dosage	36.3 % (385/1060)	24.2 % (234/968)	0.56 (0.46–0.68)	<0.001	28.8 % (305/1060)	17.1 % (166/968)	0.51 (0.41–0.63)	<0.001
Equipment to keep refrigerated medication cold if evacuating (e.g., a cool box)	45.7 % (86/188)	39.6 % (67/169)	0.78 (0.51–1.19)	0.245	29.8 % (56/188)	24.9 % (42/169)	0.78 (0.49–1.25)	0.297
Extra glasses or contact lenses	25.9 % (139/536)	32.4 % (88/272)	1.37 (0.99–1.88)	0.055	15.7 % (84/536)	26.8 % (73/272)	1.97 (1.38–2.82)	<0.001
Spare fully charged hearing aid batteries	42.9 % (99/231)	42.6 % (29/68)	0.99 (0.57–1.71)	0.975	35.1 % (81/231)	16.2 % (11/68)	0.36 (0.18–0.72)	0.004
Spare false teeth/dentures	35.9 % (55/153)	22.4 % (290/1297)	0.51 (0.36–0.73)	<0.001	31.4 % (48/153)	17.2 % (223/1297)	0.45 (0.31–0.66)	<0.001
Alternative power source for electric medical equipment (or refrigerated medication)	44.1 % (56/127)	44.1 % (184/417)	1 (0.67–1.49)	0.995	33.9 % (43/127)	28.8 % (120/417)	0.79 (0.52–1.21)	0.274
Temperature management equipment that works in a power cut (e.g., heat pads, battery operated fans)	38.6 % (34/88)	43.1 % (59/137)	1.2 (0.7–2.07)	0.510	30.7 % (27/88)	25.5 % (35/137)	0.78 (0.43–1.40)	0.401
Incontinence supplies	56.4 % (106/188)	35.0 % (42/120)	0.42 (0.26–0.67)	<0.001	43.1 % (81/188)	17.5 % (21/120)	0.28 (0.16–0.49)	<0.001
Two weeks' worth of the following:								
1) Prescribed foods, feed, or feeding supplies	38.4 % (48/125)	29.4 % (30/102)	0.67 (0.38–1.17)	0.157	28.0 % (35/125)	11.8 % (12/102)	0.34 (0.17–0.70)	0.003
2) Other specific foods (e.g., due to sensory needs) that must be refrigerated or cooked	37.9 % (53/140)	36.1 % (48/133)	0.93 (0.57–1.52)	0.762	25.7 % (36/140)	20.3 % (27/133)	0.74 (0.42–1.30)	0.290
3) Medically prescribed food supplements	41.3 % (52/126)	36.0 % (40/111)	0.80 (0.47–1.36)	0.410	24.6 % (31/126)	18.9 % (21/111)	0.72 (0.38–1.34)	0.292
4) Other medical supplies (e.g., syringes, blood sugar monitoring strips or oxygen cylinders).	51.1 % (114/223)	40.0 % (46/115)	0.64 (0.40–1.01)	0.053	35.0 % (78/223)	20.0 % (23/115)	0.46 (0.27–0.79)	0.005
5) Prescribed medications	30.6 % (475/1551)	33.8 % (124/367)	1.16 (0.91–1.47)	0.240	24.7 % (383/1551)	20.7 % (76/367)	0.80 (0.6–1.05)	0.108

Percentages are calculated by the n of people who said ‘yes’ to that question (self-describe as 'vulnerable' in a disaster/household on a PSR) against the total n with/without the resource (according to the column). Hence, 38.4 % (48/125) corresponds to 48 of the 125 people who have two weeks of prescribed food responding ‘yes’ to considering themselves 'vulnerable' in a disaster.

Odds ratios correspond to the likelihood of people without the resource responding ‘yes’ to the question (self-describe as 'vulnerable' in a disaster/household on a PSR) compared to those with the resource.

The totals for both ‘self-describe as vulnerable in a disaster’ and ‘household on a PSR’ correspond to the total n of people requiring that resource from the full sample.

Thus, the number of cases varied between analyses due to restricting the full sample (n = 5148) to only those requiring the respective resource for each row of the table.

The odds of being on a PSR was significantly lower for people who required but did not have: their medications written down (OR 0.5, CI 0.41–0.63, p < 0.001); spare hearing aid batteries (OR 0.36, CI 0.18–0.72, p = 0.004); spare false teeth/dentures (OR 0.45, 0.31–0.66, p < 0.001); incontinence supplies (OR 0.28, CI 0.16–0.49, p < 0.001); prescribed foods, feed or feeding supplies (OR 0.34, CI 0.17–0.70, p = 0.003); and other medical supplies (e.g. syringes, oxygen cylinders) (OR 0.46, CI 0.27–0.79, p = 0.005). The odds of PSR signup were significantly higher for people who required but did not have spare glasses or contact lenses (OR 1.97, CI 1.38–2.82, p < 0.001).

## 4 Discussion

Our data show that most people with characteristics associated with health inequalities in disasters neither self-describe as ‘vulnerable’ in a disaster, nor are on a PSR or any other ‘vulnerability list’.

One explanation for this, derived from Self-Categorisation Theory [16] could be that people do not self-describe as ‘vulnerable’ if they have perceived control over their circumstances in a disaster, such as by having backup supplies or alternative plans. However, our findings conflicted with this explanation. If anything, people with backup resources were more likely to consider themselves ‘vulnerable’, and for all but one 'vulnerability' marker, backup resources either reflected a higher odds of PSR signup or had no association with non-registration.

An alternative explanation is that people may be unaware of how the concept of being ‘vulnerable’ or ‘vulnerability lists’ may apply to them. The term ‘vulnerable’ is often assumed to refer to a fixed state of being for people who need ‘special supportive measures’ [7,17]. Policymakers often refer to ‘the most vulnerable’ [2,18,19], potentially prompting a process of social comparison [20] against a stereotypical ‘most vulnerable’ person who has fewer strengths and resources than you have. Low self-identification as ‘vulnerable’ may be exacerbated if perceived markers of vulnerability are not fixed states (e.g. pregnancy) or are not unusual across the general population (e.g. requiring incontinence supplies, hearing aids, etc.). Without more specific information, it may be challenging for people to understand how their circumstances meet the definition of ‘vulnerable’.

Low awareness of ‘vulnerability lists’ and their utility may also explain the observed low signup rates. In the UK, the funding of advertisements relating to PSRs has been questioned in Parliament [21]. Moreover, people may not know they are eligible for the PSR if they do not self-describe as ‘vulnerable’, since language signposting them to PSRs uses this term. The unclear utility of PSRs or similar lists may further undermine engagement. Indeed, spokespeople from UK Government departments and essential services have stated people should have “their own back-up plans” to ensure they can cope even if they are signed up to a PSR [22], and to “seek advice from their medical equipment provider and local health service provider” if they rely on essential services for medical reasons [19].

Stigma associated with the term ‘vulnerable’, and with the concept of ‘vulnerability lists’ offer another explanation for our findings. The term ‘vulnerable’ is often used as a euphemism implying that disparate risk, or health inequalities are caused by general ‘weakness’ assumed to apply to anyone of the race, age, disability status, sexual orientation or other population group facing disparate risk [8,23]. This is stigmatising [23,24]. Even if these claims were uncontroversial, people may have a more positive self-concept than of being ‘weak’ because they identify more with other aspects of their identity [25]. People may similarly prefer not to be considered as intrinsically ‘vulnerable’ by others [26]. Hesitation about being considered ‘vulnerable’ was evident during COVID-19, where some people felt “embarrassed” about being identified as CEV and refused support [27]. Even for lists that do not use the term ‘vulnerable’, such as PSRs, the concept of lists as a ‘special measure’ [28,29] may be off-putting for many who do not want to be associated with ‘charity’ or ‘special help’, preferring equal opportunities to help themselves [28].

Active opposition to the terms and interventions is also possible. Some consider the term ‘vulnerable’ to be a political weapon, and both academics and disabled journalists have argued that using it obscures discriminatory policy by implying that health inequalities are inevitable, immutable, and unaffected by public health policy [8,18]. People may veto ‘vulnerability lists’ if they consider them a barrier to accountable policymaking or effective public health transformation for inclusive health protection [22].

Finally, of course, practical considerations inhibit use of PSRs [30]. Signing up to any ‘vulnerability list’ can require paperwork, disclosure of sensitive personal information, and attentiveness to re-registering or de-registering when, for example, moving house or as medical conditions arise or resolve.

If the current system is not working, what might be a better option? Reconsidering our concept of vulnerability is an important first step. The academic literature increasingly recognises ‘vulnerability’ as not fixed but ‘situational’ and the unequal consequence of inequitable systems and treatment [7,[31], [32], [33], [34]]. In disasters, inequitable systems include inaccessible evacuation routes, relief shelters, and food distribution during recovery efforts [29]. Here, it is clear how some people would be “less able to help themselves in an emergency” [35] and face poorer health outcomes due to such systemic inequalities in “socio-economic conditions, civic and social empowerment, and access to mitigation and relief resources” [29]. Sociologists argue that people are either “made vulnerable” by non-inclusive disaster policy [31], or that everybody is ‘vulnerable’, but that health inequalities arise due to unfairly distributed opportunities for resilience [7].

Our terminology therefore needs to be reconsidered. If we recognise health inequalities as a consequence of inequitable access to coping resources and opportunities, it would be more self-explanatory and potentially less harmful [8] to use specific language describing the coping resources people need (“refrigerated medications”), or situations to be accommodated (“reduced sense of smell”). Health communications that avoid vague or euphemistic language or assumptions [8] may aid people's self-identification, and support work to reduce health disparities by highlighting unambiguously what plans or provisions could reduce disparate risk. Such transparency and clarity may also help reduce unintentional breaches of Equality duties [6].

The notion that everybody is potentially ‘vulnerable’ [7] also leads us to recognise that the perceived indicators of ‘vulnerability’ are not particularly ‘special’ or uncommon [36]. In our sample, 64.4 % of participants disclosed at least one perceived indicator of vulnerability. The characteristics we surveyed did not cover the breadth of the population eligible for PSRs, which includes anyone of pensionable age, anyone living with toddlers, anybody needing documents translated, and more [37]. PSRs may still help support people, but cannot meet the requirements of the Public Sector Equality Duty because the current configuration will miss many people [[38], [39], [40]]. This further underlines the need to accommodate everyone's needs into mainstream national emergency preparedness and response.

There are several limitations to our study. First, our data may not be representative. Although we used quotas to ensure the eventual sample was nationally representative with respect to age, gender, disability status, and social grade, the sample itself was recruited from a panel who had registered to receive market research surveys. Whether the participants represent the UK population in terms of disaster preparedness is unknown. Second, our analysis relied on self-reported data. The accuracy of self-report for our outcomes is unknown. Third, our data were collected during the summer of 2022 before a period of severe inflation in the UK. Access to resources may have since declined as people used up the supplies within their homes [18,41].

To conclude, our data suggest that most people in the UK do not consider themselves to be ‘vulnerable’ in a disaster. Official messages using the term 'vulnerable' will not effectively reach their target audiences. Most people are also not signed up to ‘vulnerability lists’. It would be imprudent to rely solely on lists, or ‘lists of lists’ going forwards.

## 5 Implications for policy and practice

• Disaster preparedness and response messages that use ‘vulnerable’ terminology will either not reach relevant people or will not align with the way they would describe themselves. Using specific, unambiguous language would be more inclusive and effective.
• Relying on the Priority Service Register or other ‘vulnerability lists’ to provide health protection to people whose requirements or circumstances are not accommodated by wider disaster response is not practical, equitable or appropriate. Therefore, public health policy cannot depend on this strategy for reducing health inequalities in practice.

Segregated measures should only be additional to wider, universal health protection policies and practices to ensure equitable access to health protection in disasters without the use of separate lists or schemes. This means that primary disaster preparedness and response efforts must accommodate diversity to promote health equity and inclusion and address health inequalities in disasters.

## Declaration of generative AI and AI-assisted technologies in the writing process

During the preparation of this work the authors used ChatGPTv3.5 in order to improve readability of the work. AI was not used to produce scientific insights, analyse or interpret data, or draw scientific conclusions. After using this tool/service, the authors reviewed and edited the content as needed and take full responsibility for the content of the publication and the originality, accuracy, and integrity of the work.

## Declaration of competing interest

This work is original and has not been published elsewhere, nor is it currently under consideration for publication elsewhere. We have no conflicts of interest to disclose.

Our second author passed away during the drafting process for this submission, so could not confirm any competing or conflicts of interest.

## References

[1] Department for Business (2022). Energy and industrial strategy. Energy emergencies executive committee storm arwen review [internet]. HM gov. https://assets.publishing.service.gov.uk/government/uploads/system/uploads/attachment_data/file/1081116/storm-arwen-review-final-report.pdf.

[2] HM Treasury (2022). https://www.gov.uk/government/news/millions-of-most-vulnerable-households-will-receive-1200-of-help-with-cost-of-living.

[3] HM Government (2022 Dec). https://www.gov.uk/government/publications/the-uk-government-resilience-framework.

[4] Civil Contingencies Secretariat (2008 Feb). https://assets.publishing.service.gov.uk/government/uploads/system/uploads/attachment_data/file/61228/vulnerable_guidance.pdf.

[5] National Audit Office (2021). Covid-19: supporting the vulnerable during lockdown [internet]. UK parliament. https://committees.parliament.uk/work/1003/covid19-supporting-the-vulnerable-during-lockdown.

[6] Women and Equalities Committee - House of Commons (2020). https://publications.parliament.uk/pa/cm5801/cmselect/cmwomeq/1050/105002.htm.

[7] Gabel F, Krüger M, Kuran C, Morsut C. Bridging the Gap between Vulnerable Groups and Vulnerable Situations: Towards an Integrative Perspective New Assessment on Vulnerability for Disaster Risk Reduction. GAR2022 Contributing Paper. Geneva: United Nations Office for Disaster Risk Reduction. www.undrr.org/gar2022 [Internet]. UNDRR; 2022 Apr [cited 2023 Oct 24]. Available from: http://dx.doi.org/.

[8] Katz A.S., Hardy B.J., Firestone M., Lofters A., Morton-Ninomiya M.E. (2020 Oct 19). Vagueness, power and public health: use of ‘vulnerable‘ in public health literature. Crit. Publ. Health.

[9] Equality Act 2010: guidance [Internet]. Gov.uk. [cited 2023 Mar 3]. Available from: https://www.gov.uk/guidance/equality-act-2010-guidance.

[10] (1998). Human rights Act 1998 [internet]. Sect. Chapter 42 nov 9. https://www.legislation.gov.uk/ukpga/1998/42/schedule/1.

[11] Equality and Human Rights Commission (2020). Equality and Human Rights Commission.

[12] Park N. (2021). https://www.ons.gov.uk/peoplepopulationandcommunity/populationandmigration/populationestimates/datasets/populationestimatesforukenglandandwalesscotlandandnorthernireland.

[13] Park N. (2021). https://www.ons.gov.uk/peoplepopulationandcommunity/populationandmigration/populationestimates/timeseries/ukpop/pop.

[14] Office for National Statistics (2022). https://statistics.ukdataservice.ac.uk/dataset/approximated-social-grade-household-reference-persons-2011.

[15] Department for Work and Pensions (2022). https://www.gov.uk/government/statistics/family-resources-survey-financial-year-2020-to-2021/family-resources-survey-financial-year-2020-to-2021.

[16] Oakes P.J., Alexander Haslam S. (1994). https://play.google.com/store/books/details?id=KvhnQgAACAAJ.

[17] Kailes J.I., Enders A. (2007 Mar 1). Moving beyond “special needs”: a function-based framework for emergency management and planning. Journal of Disability Policy Studies [Internet].

[18] Ryan F. (2022 Nov 29). Disabled children can't afford to use their ventilators this winter. Politicians need to face reality. The Guardian [Internet].

[19] UK Parliament (2022). Question for department for business, energy and industrial strategy [internet]. Written questions, answers and statements - Medical Equipment: Electricity.

[20] De Cremer D. (2001 Jun 1). Perceptions of group homogeneity as a function of social comparison: the mediating role of group identity. Curr Psychol [Internet].

[21] UK Parliament (2022). Question for department for business, energy and industrial strategy [internet]. Written questions, answers and statements - Energy Supply: Advertising.

[22] Pring J. (2022). Winter blackouts: disabled people must draw up their own back-up plans, say ministers [Internet]. Disability News Service.

[23] Avila C.J. (2020). “Vulnerable” is a public health euphemism we need to stop using [Internet]. The Incidental Economist.

[24] The Lancet Infectious Diseases (2022 Aug). Reaching the vulnerable without stigma. Lancet Infect Dis [Internet].

[25] Turner J.C., Lawler E. (1985). Advances in Group Processes [Internet].

[26] Social model of disability [Internet]. Scope. [cited 2023 Mar 3]. Available from: https://www.scope.org.uk/about-us/social-model-of-disability/.

[27] Bruce R., Dunkley N., Mathers A., Verlot M., Soorenian A., Olsen J. (2021). https://assets.publishing.service.gov.uk/media/614893f6d3bf7f05b5a903a9/CCS118_CCS0721976312-002_Quasi-ethnographic_report_edits_Large_Print_Report_2.pdf.

[28] United Nations (2006). Convention on the rights of persons with disabilities. Vol. 61, united nations. Convention on the Rights of Persons with Disabilities.

[29] Disability-Inclusive Disaster Risk Reduction and Emergency Situations [Internet]. [cited 2023 Mar 15]. Available from: https://www.un.org/development/desa/disabilities/issues/disability-inclusive-disaster-risk-reduction-and-emergency-situations.html.

[30] Civil Protection Team (2015 May). https://www.gloshospitals.nhs.uk/media/documents/Vulnerable_People_Plan.pdf.

[31] Tierney K. (2019). https://play.google.com/store/books/details?id=HY2ZDwAAQBAJ.

[32] Munari S.C., Wilson A.N., Blow N.J., Homer C.S.E., Ward J.E. (2021 Jun). Rethinking the use of ‘vulnerable’. Aust N Z J Public Health [Internet].

[33] Williams D.R., Mohammed S.A. (2013 Aug 1). Racism and health I: pathways and scientific evidence. Am Behav Sci [Internet].

[34] Lofters A., O'Campo P., O'Campo P., Dunn J.R. (2012). Rethinking Social Epidemiology: towards a Science of Change [Internet].

[35] Cabinet Office (2013). https://www.gov.uk/guidance/preparation-and-planning-for-emergencies-responsibilities-of-responder-agencies-and-others.

[36] Disability Unit of the UK Government Cabinet Office (2021). https://www.gov.uk/government/publications/inclusive-communication/portraying-disability.

[37] The PSR - The Priority Services Register (PSR) is a free UK wide service which provides extra advice and support, including when there's an interruption to your electricity or gas supply - PSR [Internet]. [cited 2023 Oct 17]. Available from: https://www.thepsr.co.uk/.

[38] Equality and Human Rights Commission (2023). https://www.equalityhumanrights.com/en/advice-and-guidance/public-sector-equality-duty.

[39] Equality and Human Rights Commission (2022). Equality and Human Rights Commission.

[40] (2021). Equality and Human Rights Commission.

[41] Inclusion Scotland (2022). https://inclusionscotland.org/home-page-news/cost-of-living-crisis-hits-disabled-people-hard-new-report-published.

