# Supplementary material for: Reducing health inequalities in disasters: A cross-sectional study of the viability of ‘vulnerability’ terminology and of priority lists in the UK
Source: Public Health Pract (Oxf). 2024 Dec 20;9:100564. doi: 10.1016/j.puhip.2024.100564 (PMC11795149; doi:10.1016/j.puhip.2024.100564)
Supplement: Multimedia component 1 [file mmc1.docx]

# Final Questionnaire

The Disaster Perceptions and Needs Index // Extreme Events Resilience Index

Classified: Private

| **PROGRAMMING GUIDELINES** | | |
| --- | --- | --- |
| **SURVEY NAME TO APPEAR ON URL** | Marketing survey |  |
| **SAMPLE SOURCE** | Savanta panels |  |
| **BRANDING** | Standard Savanta branding |  |
| **BACK BUTTON** | No (disabled) |  |
| **PROGRESS BAR** | Yes |  |
| **LANGUAGES** | English |  |
| **QUOTAS** | UK nat rep (age, gender, region, SEG, disability/chronic illness) |  |
| **ESTIMATED TOTAL COMPLETES** | N=5,000 |  |
| **IN-SURVEY REDIRECTS** | None |  |
| **SCREEN OUT REDIRECT** | Panel links |  |
| **END REDIRECT** | Client’s website |  |


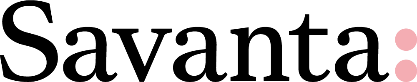


Welcome! Thank you for your interest in this survey about disasters.

This research project is being conducted by researchers at King’s College London and the United Kingdom Health Security Agency. Before taking part, it is important for you to understand why the research is being done and what your participation will involve. Please take time to read the following information carefully and discuss it with others if you wish.

**Measuring disaster support needs**

We would like to invite you to participate in this survey about support needs and disasters. You should only participate if you want to; choosing not to take part will not disadvantage you in any way. Before you decide whether you want to take part, it is important for you to understand why the research is being done and what your participation will involve.

**What is the purpose of this research?**

The aim of this research is to identify what support households across the UK might need in a disaster. This includes investigating what needs people will have in disasters, and who might be disproportionately affected by the health risks of a disaster. We will be investigating what people think of disasters in general, as well as how people think about the risks associated with different types of disaster. We’ll also be looking to whether the way people describe themselves and prepare for disasters match the way disaster planners in the UK are currently working.

**Who can take part?**

You can take part in the survey if you are 18 or older, understand English and live in the UK.

**What will I be asked to do?**

If you decide to take part, you will be asked a number of questions about your background in general, your views of disasters in general, what health-related needs you or your household might have in a disaster, any preparations you have in place in case of a disaster, what you think you might do in a disaster, and some questions about whether you have previously experienced certain disasters.

Your responses about your background will be combined with those of other respondents to see if there are any common themes in who might need more support in disasters, and whether there are any differences in the way people view disasters between people from different backgrounds.

Some of the questions in this study may seem sensitive as they include questions about what your needs may be in a disaster, including medical or other care needs. There are also some questions about demographics. There is the option to select ‘prefer not to say’ for all of these.

The survey will take between 20-25 minutes to complete and we would like you to complete it in one sitting. People who begin the study, but who are ‘screened out’ in the first few questions will not be shown the rest of the questionnaire. The likelihood of this is very low, and would only occur if we have already received a disproportionate number of responses from people in your area – as we are seeking as nationally representative a sample as possible. If you do not complete the questionnaire in full your data will not be used in the research.

**Are there any risks associated with participation?**

There are no known significant risks associated with this study. There is a small chance that you may find some of the content of the task unpleasant. However, all the content used is the type of information that people generally encounter in everyday life, for example in newspapers or on television.

Please note that you will be asked questions about your plans for disasters and any health needs you may have. If you feel uncomfortable about health questions you can answer ‘prefer not to say’.

If you feel it is necessary, you can stop the session entirely by closing the browser at any time. You can also contact researchers if you feel upset or concerned about anything after the session.

Please note you have the right to decline or withdraw from the study without reason or penalty.

If you exit the survey early, answers that you have given will not be recorded as part of the research. Once you submit the survey, it will no longer be possible to withdraw from the study because the data will be fully anonymous.

**Confidentiality – who will know I am taking part in the study?**

All the information that we collect will be kept strictly confidential and in accordance with UK data protection laws (including the UK General Data Protection Regulation (UK GDPR) and the Data Protection Act 2018). We will not pass on any details that might identify you to the researchers at King’s College London, or to anyone else.

**What will happen to my response?**

You will submit your answers to Savanta in the normal way, under the terms and conditions you agreed to when joining their panel. Savanta will then provide a spreadsheet of responses to our research team. This will be anonymous. This means that nobody, including the researchers, will be aware of your identity, and that nobody will be able to connect you to the answers you provide, even indirectly. The research team hope to publish the findings of this work in a scientific journal, and may make the anonymous spreadsheet publicly available as part of this process so that other scientists can check it. The data controller for this project will be King’s College London (KCL). This data may also be used by other researchers for secondary analysis. Research is a task that the University carries out in the public interest. Your data will be processed under the terms of UK data protection law (including the UK General Data Protection Regulation (UK GDPR) and the Data Protection Act 2018).

**What if I change my mind about taking part?**

You are free to withdraw at any point before submitting your response by closing the survey window. Because the survey is anonymous, we cannot identify your response to remove it after you have submitted it as we will not know which one is yours. However, if you close the survey before completing it, none of your responses will be recorded.

**What will happen to the results of the study?**

The team from King’s College London hope to publish the results of this study in a scientific journal. The results of the project will be summarised in Poppy Ellis Logan’s PhD thesis, which will be published after 2024 and accessible via the King’s Research Portal. The anonymous results from this survey may also be shared with other researchers and policy makers who are interested in this work in the future.

**Who has funded and approved the research?**

This work is funded by King’s College London. For more information about our funding and research, please visit http://epr.hpru.nihr.ac.uk/. It has been approved by King’s College London’s Health Faculties Research Ethics Committee (Reference: HR-21/22-28388). The survey is being run in accordance with the Market Research Society Code of Conduct and is part of a PhD project at King’s College London.

**Do I have to take part?**

No. It is up to you to decide whether to take part or not. If you decide to take part you are free to withdraw from the survey at any time by simply closing the browser window on your computer. Once you have completed and submitted the survey you can no longer withdraw your data.

**What happens now?**

If you would like to take part, please click on the arrow below to begin. Completing the survey implies that you give consent to participate. If you would like to take some time to consider your decision before taking part, you can always close the survey now and come back later.

**How can I find out about the results of the study?**

The results of the study will be published on the Emergency Preparedness and Response Health Protection Research Unit website (http://epr.hpru.nihr.ac.uk/). Alternatively, anyone can contact the lead researcher (Poppy Ellis Logan: poppy.ellis_logan@kcl.ac.uk) directly and she will let share the results of the study in due course.

**Who should I contact for further information?**

If you would like more details about the survey, please contact the lead researcher at King’s College London (Poppy Ellis Logan). Her details are:

Poppy Ellis Logan (she/her)

King’s College London

Department of Psychological Medicine

Weston Education Centre, Cutcombe Road

London SE5 9RJ

e-mail: [poppy.ellis_logan@kcl.ac.uk](mailto:poppy.ellis_logan@kcl.ac.uk)

**What if I have further questions, or if something goes wrong?**

If this study has harmed you in any way or if you wish to make a complaint about the conduct of the study you can contact King's College London using the details below for further advice and information:

Professor James Rubin

Psychological Medicine Department

Weston Education Centre, Cutcombe Road

London SE5 9RJ

Phone: +44(0)20 7848 5684

e-mail: gideon.rubin@kcl.ac.uk

For authentication and quality purposes, cookies may be used to collect your personal data during the course of the survey. See our [cookie policy](https://www.savanta.com/cookies) and [privacy policy](https://savanta.com/privacy-policy) for additional details.

ASK ALL

CONSENT. If you would like to take part in the study, please confirm the following.

(If you do not want to take part in the study, you do not need to do anything.)

Please select all that apply

MULTICODE

| I consent to the processing of my personal information for the purposes of this research study. I understand that such information will be treated as strictly confidential and handled in accordance with the provisions of UK data protection law (including the UK General Data Protection Regulation (UK GDPR) and the Data Protection Act 2018) | 1 | IF DO NOT SELECT, SCREEN OUT |
| --- | --- | --- |
| I understand that my information may be reviewed for research, monitoring and audit purposes by responsible individuals from King’s College London. I understand that they will not be able to identify me from my response. | 2 | IF DO NOT SELECT, SCREEN OUT |
| I understand that the data from this survey may also be shared with other researchers or policymakers, but never in an identifiable format and always in accordance with UK data protection law as specified above. | 3 | IF DO NOT SELECT, SCREEN OUT |
| I understand that confidentiality and anonymity will be maintained, and it will also not be possible to identify me in any research outputs | 4 | IF DO NOT SELECT, SCREEN OUT |
| I have read the information presented above in full, understand what the research study involves and have no outstanding questions about it. | 5 | IF DO NOT SELECT, SCREEN OUT |
| I confirm that the research project named above has been explained to me to my satisfaction and I agree to take part in the study. | 6 | IF DO NOT SELECT, SCREEN OUT |
| I consent voluntarily to participate in this project and understand that I can withdraw at any point during the survey by closing the page, and that my responses will not be recorded if I do this before completing the survey. | 7 | IF DO NOT SELECT, SCREEN OUT |

# Screening and profiling

ASK ALL

AGE. How old are you?

Please move the slider until it shows your age in the box on the left.

SLIDER

Min 0 ---- || ---- Max 150

Terminate if < 18

**DUMMY VARIABLE**

dAGE.

Dummy Age Bands

RECODE AGE INTO...

SINGLE CODE

| 18-24 | 1 |  |
| --- | --- | --- |
| 25-34 | 2 |  |
| 35-44 | 3 |  |
| 45-54 | 4 |  |
| 55-64 | 5 |  |
| 65-74 | 6 |  |
| 75-84 | 7 |  |
| 85+ | 8 |  |

ASK ALL

GENDER. In which of the following ways do you identify?

Please select one option

SINGLE CODE

| Female | 2 |  |
| --- | --- | --- |
| Male | 1 |  |
| I identify in another way | 98 | FIX OE |
| Prefer not to say | 96 | FIX |

ASK ALL

GENDER_2. Are you transgender, agender, or gender diverse?

Please select one option

SINGLE CODE

| Yes | 1 |  |
| --- | --- | --- |
| No | 2 |  |
| I identify in a way other than what is listed here | 98 | FIX OE |
| Prefer not to say | 96 | FIX |

ASK ALL

UK_REGION. Where do you live?

Please select one option

SINGLE CODE

| Northern Ireland | 1 |  |
| --- | --- | --- |
| Scotland | 2 |  |
| North-West England | 3 |  |
| North-East England | 4 |  |
| Yorkshire & Humberside | 5 |  |
| Wales | 6 |  |
| West Midlands | 7 |  |
| East Midlands | 8 |  |
| South-West England | 9 |  |
| South-East England | 10 |  |
| Eastern England | 11 |  |
| London | 12 |  |
| Channel Islands | 13 | SCREEN OUT |
| Outside the UK | 14 | SCREEN OUT |

ASK ALL

OCCUPATION. Please indicate which one of the following best describes the profession of the highest income earner in your household.

*Please select one option*

SINGLE CODE

| High managerial, administrative or professional e.g. doctor, lawyer, medium / large company director (50+ people) | 1 |  |
| --- | --- | --- |
| Intermediate managerial, administrative or professional e.g. teacher, manager, accountant | 2 |  |
| Supervisor, administrative or professional e.g. police officer, nurse, secretary, self-employed | 3 |  |
| Skilled manual worker e.g. mechanic, plumber, electrician, lorry driver, train driver | 4 |  |
| Semi-skilled or unskilled manual worker e.g. waiter, factory worker, receptionist, labourer | 5 |  |
| House-wife / house-husband | 6 |  |
| Unemployed | 7 |  |
| Student | 8 |  |
| Retired | 9 |  |

ASK IF OCCUPATION = RETIRED [CODE 9]

OCCUPATIONRETIRED. Which of the following best describes the previous occupation of the highest income earner in your household before retirement?

*Please select one option*

SINGLE CODE

| High managerial, administrative or professional e.g. doctor, lawyer, medium / large company director (50+ people) | 1 |  |
| --- | --- | --- |
| Intermediate managerial, administrative or professional e.g. teacher, manager, accountant | 2 |  |
| Supervisor, administrative or professional e.g. policeman, nurse, secretary, self-employed | 3 |  |
| Skilled manual worker e.g. mechanic, plumber, electrician, lorry driver, train driver | 4 |  |
| Semi-skilled or unskilled manual worker e.g. waiter, factory worker, receptionist, labourer | 5 |  |
| House-wife / house-husband | 6 |  |
| Unemployed | 7 |  |
| Student | 8 |  |

**DUMMY VARIABLE**

dSEG DUMMY SEG

RECODE OCCUPATION INTO

SINGLE CODE

| A | 1 | OCCUPATION/OCCUPATIONRETIRED = 1 |
| --- | --- | --- |
| B | 2 | OCCUPATION/OCCUPATIONRETIRED = 2 |
| C1 | 3 | OCCUPATION/OCCUPATIONRETIRED = 3 or 8 |
| C2 | 4 | OCCUPATION/OCCUPATIONRETIRED = 4 |
| D | 5 | OCCUPATION/OCCUPATIONRETIRED = 5 |
| E | 6 | OCCUPATION/OCCUPATIONRETIRED = 6 or 7 or 9 |

# Household

ASK ALL

CHILDREN. Are you a parent, legal guardian, or other carer, to any children aged 17 or under?

Please select one option

SINGLE CODE

| Yes | 1 |  |
| --- | --- | --- |
| No | 2 |  |
| Prefer not to say | 96 |  |

ASK IF CODE 1 FOR CHILDREN

CHILDREN_AGE. Which of the following age groups do the children you care for fall into?

Please select all that apply

MULTICODE

| 0-2 years | 1 |  |
| --- | --- | --- |
| 3-6 years | 2 |  |
| 7-12 years | 3 |  |
| 13-15 years | 4 |  |
| 16-17 years | 5 |  |
| Prefer not to say | 96 | FIX EXCLUSIVE |

ASK IF CHILDREN = 1 (Yes)

SCHOOL. Does your child/do the children aged 17 or under that you care for attend college, school, nursery or any other educational facility?

Please select one option

SINGLE CODE

| Yes | 1 |  |
| --- | --- | --- |
| No | 2 |  |

ASK IF SCHOOL = 1 (Yes)

FSM. Does the child / do the children you care for receive Free School Meals?

Please select one option

SINGLE CODE

| Yes | 1 |  |
| --- | --- | --- |
| No | 2 |  |
| I don’t know | 3 |  |
| Prefer not to say | 4 |  |

ASK IF CHILDREN = 1 (Yes)

CHILDREN_NUM. How many children aged 17 or under **live with you** most of the time?

Type your answer below

OPEN END, NUMERIC, MIN 0 – MAX 50

|  |
| --- |

ASK ALL

CARER. Do you look after or care for anyone (e.g. friends, family, neighbours) aged 18 or over? This could be as a paid carer, or regular unpaid help with everyday tasks such as getting groceries, translating things into English, or keeping them company.

Please select one option

SINGLE CODE

| Yes | 1 |  |
| --- | --- | --- |
| Sometimes | 2 |  |
| No | 3 |  |
| Prefer not to say | 96 |  |

ASK IF CARER = 1-2 (Yes or Sometimes)

CARER_NUM. How many people aged 18 or over do you look after or care for?

Type your answer below

OPEN END, NUMERIC, MIN 0 – MAX 1000

|  |
| --- |

CARER = 1-2 OR ‘CHILDREN’=1

CARER_HOUSEHOLD. Do you look after or care for anyone (including children) who lives outside of your household?

Please select one option

SINGLE CODE

| Yes | 1 |  |
| --- | --- | --- |
| Some of the people I look after do live in my household, but not all | 2 | SHOW IF THEY ENTER ‘2’ OR MORE AT CARER_NUM OR CHILDREN_NUM |
| No | 3 |  |
| Prefer not to say | 96 |  |

ASK IF CARER = 1-2 (Yes or Sometimes)

CARER_AGE. Which of the following age groups do the adults you care for fall into?

Please select all that apply

MULTICODE, SINGLECODE IF ENTER ‘1’ AT CARER_NUM

| 18-24 | 1 |  |
| --- | --- | --- |
| 25-34 | 2 |  |
| 35-44 | 3 |  |
| 45-54 | 4 |  |
| 55-64 | 5 |  |
| 65-74 | 6 |  |
| 75-84 | 7 |  |
| 85+ | 8 |  |
| Prefer not to say | 96 | FIX EXCLUSIVE |

ASK IF CARER_HOUSEHOLD = 1-2 (Yes or Some)

CARER_DISASTER. If you and your household had to leave your neighbourhood because of a disaster or emergency, would someone else need to look after the person or people you care for **outside** your household?

Please select one option

SINGLE CODE

| Yes, and I know who would do it | 1 |  |
| --- | --- | --- |
| Yes, but I don’t know who would do it | 2 |  |
| No | 3 |  |
| Prefer not to say | 96 |  |

ASK ALL

HOUSEHOLD_SIZE. How many people live in your household including yourself?

Please select one option

SINGLE CODE

| 1 | 1 |  |
| --- | --- | --- |
| 2 | 2 |  |
| 3-4 | 3 |  |
| 5-6 | 4 |  |
| 7 or more | 5 |  |
| Prefer not to say | 96 |  |

ASK ALL

PETS. How many pets, if any, do you have?

Type your answer below

OPEN END, NUMERIC, MIN 0, MAX 50

|  |
| --- |

ASK ALL

ANIMALS. How many animals, if any, do you, or your business / charity own? Please include in this figure animals owned as livestock, as well as any animals whose wellbeing you are legally responsible for. Do not include pets.

Type your answer below

OPEN END, NUMERIC, MIN 0

|  |
| --- |

# Demographics

ASK ALL

ETHNICITY. How would you describe your ethnic origin?

Please select one option

SINGLE CODE. RANDOMISE OVERALL CATEGORIES IN GREY. FIX SUB-CATEGORIES. FIX ‘Other ethnic group’.

| White |  | RANDOMISE |
| --- | --- | --- |
| White English / Welsh / Scottish / Northern Irish / British | 1 |  |
| White Irish | 2 |  |
| Gypsy or Irish Traveller | 3 |  |
| Any other White background | 4 |  |
| Mixed/Multiple ethnic groups |  | RANDOMISE |
| Mixed White and Black Caribbean | 5 |  |
| Mixed White and Black African | 6 |  |
| Mixed White and Asian | 7 |  |
| Any other mixed/multiple ethnic background | 8 |  |
| Asian or Asian British |  | RANDOMISE |
| Indian | 9 |  |
| Pakistani | 10 |  |
| Bangladeshi | 11 |  |
| Chinese | 12 |  |
| Any other Asian background | 13 |  |
| Black or Black British |  | RANDOMISE |
| Black African | 14 |  |
| Black Caribbean | 15 |  |
| Black British or any other Black background | 16 |  |
| Other ethnic group |  | RANDOMISE |
| Arab | 17 |  |
| Not listed (please specify) | 98 | FIX OE |
| Prefer not to say | 96 |  |

ASK ALL

RELIGION. What is your religion?

Please select one option

SINGLE CODE

RANDOMISE

| Christian (includes Church of England, Catholic, Protestant, and all other Christian denominations) | 1 |  |
| --- | --- | --- |
| Muslim | 2 |  |
| Hindu | 3 |  |
| Jew | 4 |  |
| Sikh | 5 |  |
| Buddhist | 6 |  |
| Other (please specify) | 98 | FIX OE |
| None | 99 | FIX EXCLUSIVE |
| Prefer not to say | 96 | FIX EXCLUSIVE |

ASK ALL

EMP_STATUS. Which of the following best describes your current working status?

Please select one option that best describes the majority of your time

SINGLE CODE

| Working full time - working 30 hours per week or more – or on leave from a full-time job. | 1 |  |
| --- | --- | --- |
| Working part time – guaranteed hours, working up to 29 hours per week, or on leave from a part-time job. | 2 |  |
| Working part time – zero hours contract | 3 |  |
| Not working but seeking work | 4 |  |
| Not working and not seeking work, **due to** long-term illness or disability | 5 |  |
| Not working and not seeking work, for reasons **other than** long term illness or disability |  |  |
| Student | 6 |  |
| Retired on a state pension only | 7 |  |
| Retired with a private pension | 8 |  |
| Stay-at-home spouse/partner | 9 |  |
| Unpaid carer | 10 |  |
| Unpaid volunteer | 11 |  |
| Not listed (please specify) | 98 | FIX OE |
| Prefer not to say | 96 | FIX |

ASK IF EMP_STATUS = 1-3

INDUSTRY_LONG. Which sector do you work in?

Please select one option

SINGLE CODE

| Accommodation and hospitality | 1 |  |
| --- | --- | --- |
| Accounting and auditing services | 2 |  |
| Administrative and support services | 3 |  |
| Agriculture, forestry and fishing | 4 |  |
| Arts, entertainment and recreation | 5 |  |
| Construction | 6 |  |
| Education - primary or secondary school | 7 |  |
| Education - other (e.g. college, university etc.) | 8 |  |
| Electricity, gas, steam and air conditioning supply | 9 |  |
| Finance and insurance | 10 |  |
| General banking | 11 |  |
| Healthcare (includes dentists and pharmaceuticals) | 12 |  |
| IT and telecoms | 13 |  |
| Legal sector | 14 |  |
| Manufacturing | 15 |  |
| Market research | 16 | SCREEN OUT |
| Mining | 17 |  |
| Property and real estate | 18 |  |
| Public administration and defence | 19 |  |
| Retail and wholesale | 20 |  |
| Transport and logistics | 21 |  |
| Water supply, sewerage and waste management | 22 |  |
| Other (please specify) | 98 | FIX OE |

ASK ALL

QUALIFICATION What is the highest level of educational or professional qualification you have received? Please type in and select from the options below.

Type your answer below

OPEN END

|  |
| --- |

| No formal qualifications | 1 |  |
| --- | --- | --- |
| Youth training certificate/skillseekers | 2 |  |
| Recognised trade apprenticeship | 3 |  |
| Clerical and commercial | 4 |  |
| City & Guilds certificate | 5 |  |
| City & Guilds certificate – advanced | 6 |  |
| ONC | 7 |  |
| CSE grades 2-5 | 8 |  |
| CSE grade 1, GCE O level, GCSE, School Certificate | 9 |  |
| Scottish Ordinary/Lower Certificate | 10 |  |
| GCE A level or Higher Certificate | 11 |  |
| Scottish Higher Certificate | 12 |  |
| NVQ | 13 |  |
| Nursing or midwifery qualification (e.g. SEN, SRN, SCM, RGN) | 14 |  |
| Teaching qualification (not degree) | 15 |  |
| University diploma | 16 |  |
| University or CNAA first degree (e.g. BA, BSc, BEd) | 17 |  |
| University or CNAA higher degree (e.g. MSc, PhD) | 18 |  |
| Other technical, professional or higher qualification | 19 |  |
| Don’t know | 97 | FIX |
| Prefer not to say | 96 | FIX |

ASK ALL

KEY_WORKER. Were you a key worker during the early stages of the pandemic (e.g. 2020)?

Please select one option

SINGLE CODE

| Yes | 1 |  |
| --- | --- | --- |
| No | 2 |  |
| Don’t know | 97 |  |
| Prefer not to say | 96 |  |

ASK ALL

ProvidePostcode. Are you happy to provide us with your postcode?

*This information will be used for statistical purposes only, for example, to link you to a particular UK region (London, North West, Wales, etc.). The postcode information itself will* ***not*** *be shared with King’s College London or the UKHSA. Asking for your postcode saves you time and helps us to report more accurate information. All answers will be treated entirely anonymously, and postcode information will not be used for any other purpose.*

Please select one option

SINGLE CODE

| Yes | 1 |  |
| --- | --- | --- |
| No | 2 |  |

ASK IF ProvidePostcode = 1 (Yes)

Postcode. What is your postcode?

*Please note: This question may be considered personal. We would like to remind you that your participation is strictly voluntary and that your responses are used for research purposes only. The answers that you provide will be presented in aggregate form and none of them will be linked back to you in any way. All data will be collected and processed in adherence to the Market Research Society’s Code of Conduct and the General Data Protection Regulation (GDPR).*

Type your answer below

OPEN END

| OPEN | 1 |  |
| --- | --- | --- |

ASK ALL

LIVING_STATUS. Which of the following best describes your current living situation in the UK?

Please select one option

SINGLE CODE

| Homeowner (with a mortgage) | 1 |  |
| --- | --- | --- |
| Homeowner (without a mortgage) | 2 |  |
| Living at home with parents | 3 |  |
| Tenant (in private housing) | 4 |  |
| Tenant (living in social / council housing or a housing association) | 5 |  |
| Tenant (student halls) | 6 |  |
| Tenant (supported accommodation) | 7 |  |
| In temporary accommodation / hostels | 8 |  |
| Homeless | 9 |  |
| Not listed (please specify) | 98 | FIX OE |

ASK ALL

INCOME. What is your annual household income, before tax and deductions?

Please select one option

SINGLE CODE

| Less than £20,000 | 1 |  |
| --- | --- | --- |
| £20,000 - £29,999 | 2 |  |
| £30,000 - £39,999 | 3 |  |
| £40,000 - £49,999 | 4 |  |
| £50,000 - £59,999 | 5 |  |
| £60,000 - £69,999 | 6 |  |
| £70,000 - £79,999 | 7 |  |
| £80,000 or more | 8 |  |
| Don’t know | 97 |  |
| Prefer not to say | 96 |  |

# Household self-description

ASK ALL

BENEFITS. Do you or anyone in your household receive any of the following benefits:

Please select all that apply

MULTICODE, RANDOMISE

| Personal Independence Payment (PIP) | 1 |  |
| --- | --- | --- |
| Disability Living Allowance (DLA) | 2 |  |
| Independent Living Fund (ILF) | 3 |  |
| Attendance Allowance | 4 |  |
| Carer’s Allowance | 5 |  |
| Carer’s Credit | 6 |  |
| Universal Credit | 7 |  |
| Other disability-related benefit | 98 | FIX OE |
| Don't know | 97 | FIX EXCLUSIVE |
| Prefer not to say | 96 | FIX EXCLUSIVE |
| None of these | 99 | FIX EXCLUSIVE |

ASK ALL

VULNERABLE_SELF. Would you consider yourself to be vulnerable during a disaster?

Please select one option

SINGLE CODE

| Yes | 1 |  |
| --- | --- | --- |
| No | 2 |  |
| Maybe | 3 |  |
| Prefer not to say | 96 |  |
| Don’t know | 97 |  |

ASK ALL

VULNERABLE_HOUSEHOLD. Would you consider anyone else in your household to be vulnerable during a disaster?

Please select one option

SINGLE CODE

| Yes | 1 |  |
| --- | --- | --- |
| No | 2 |  |
| Maybe | 3 |  |
| Prefer not to say | 96 |  |
| Don’t know | 97 |  |
| Not applicable | 99 |  |

ASK IF CARER_HOUSEHOLD = 1-2 (Yes or Some)

VULNERABLE_CARE. Would you consider anyone you look after or care for **outside** your household for to be vulnerable during a disaster?

Please select one option

SINGLE CODE

| Yes | 1 |  |
| --- | --- | --- |
| No | 2 |  |
| Maybe | 3 |  |
| Prefer not to say | 96 |  |
| Don’t know | 97 |  |

ASK ALL

DISABILITY. Would you refer to yourself as disabled?

Please select one option

SINGLE CODE

| Yes | 1 |  |
| --- | --- | --- |
| No | 2 |  |
| Prefer not to say | 96 |  |
| Don’t know | 97 |  |

ASK ALL

DISABILITY_HOUSEHOLD. Would you refer to anyone else in your household as disabled?

Please select one option

SINGLE CODE

| Yes | 1 |  |
| --- | --- | --- |
| No | 2 |  |
| Prefer not to say | 96 |  |
| Don’t know | 97 |  |
| Not applicable | 99 |  |

ASK IF CARER_HOUSEHOLD = 1-2 (Yes or Some)

DISABILITY_CARE. Would you refer to anyone you look after or care for **outside** your household as disabled?

Please select one option

SINGLE CODE

| Yes | 1 |  |
| --- | --- | --- |
| No | 2 |  |
| Prefer not to say | 96 |  |
| Don’t know | 97 |  |

# Part 1

## Perceptions

ASK ALL

Q1. Different parts of the country are more likely to experience certain events than other parts of the country. How likely is it that your neighbourhood would experience the following in the next three years? Please provide your best guess.

Please select one option for each answer

SINGLE CODE

CAROUSEL, RANDOMISE STATEMENTS, FLIP SCALE

Answer Options

| Very likely | 5 |  |
| --- | --- | --- |
| Likely | 4 |  |
| Neither likely nor unlikely | 3 |  |
| Unlikely | 2 |  |
| Very unlikely | 1 |  |
| Don't know | 97 |  |
| Prefer not to say | 96 |  |

Statements

| Widespread electricity failures | 1 |  |
| --- | --- | --- |
| Low temperatures | 2 |  |
| River flooding | 3 |  |
| Cyber attacks | 4 |  |
| Coastal flooding | 5 |  |
| Storms | 6 |  |
| Heatwaves | 7 |  |
| Volcanic eruptions | 8 |  |
| Nuclear industrial accident | 9 |  |
| Another pandemic | 10 |  |
| War | 11 |  |

ASK ALL

Q2. If the following were to happen to you in the next three years, how severe would the impact be for you?

Please select one option for each answer

SINGLE CODE

CAROUSEL, RANDOMISE STATEMENTS, FLIP SCALE

Answer Options

| Extremely severe impact | 5 |  |
| --- | --- | --- |
| Severe impact | 4 |  |
| High impact | 3 |  |
| Low impact | 2 |  |
| No impact | 1 |  |
| Don't know | 97 |  |
| Prefer not to say | 96 |  |

Statements

| Widespread electricity failures | 1 |  |
| --- | --- | --- |
| Low temperatures | 2 |  |
| River flooding | 3 |  |
| Cyber attacks | 4 |  |
| Coastal flooding | 5 |  |
| Storms | 6 |  |
| Heatwaves | 7 |  |
| Volcanic eruptions | 8 |  |
| Nuclear industrial accident | 9 |  |
| Another pandemic | 10 |  |
| War | 11 |  |

## Prevalence Needs

INFO SCREEN:

The following questions will help us understand specific needs you might have in a disaster. **If you sometimes have these needs (e.g. due to a health condition that comes and goes), please answer “yes”.**

ASK ALL

Q3. Are you pregnant?

Please select one option

SINGLE CODE

| Yes | 1 |  |
| --- | --- | --- |
| No | 2 |  |
| Prefer not to say | 96 |  |

ASK ALL

Q4. Are you, or a member of your household, breastfeeding an infant?

Please select one option

SINGLE CODE

| Yes | 1 |  |
| --- | --- | --- |
| No | 2 |  |
| Prefer not to say | 96 |  |

ASK ALL

Q5. Are you, or a member of your household, formula feeding an infant?

Please select one option

SINGLE CODE

| Yes | 1 |  |
| --- | --- | --- |
| No | 2 |  |
| Prefer not to say | 96 |  |

ASK ALL

Q6A. Would you describe yourself as either d/Deaf or likely to experience significant difficulty hearing?

Please select one option

SINGLE CODE

| Yes | 1 |  |
| --- | --- | --- |
| Yes, but not when using hearing aids | 2 |  |
| No | 3 |  |
| Prefer not to say | 96 |  |

ASK IF ‘CARER’=1-2 OR ‘CHILDREN’=1

Q6B. Would you describe anyone you look after or care for as either d/Deaf or likely to experience significant difficulty hearing?

Please select one option

SINGLE CODE

| Yes | 1 |  |
| --- | --- | --- |
| Yes, but not when using hearing aids | 2 |  |
| No | 3 |  |
| Don’t know | 97 |  |
| Prefer not to say | 96 |  |

ASK ALL

Q7A. Do you have any other condition(s) that may stop you from hearing alarms or other noise alerts (such as a fire bell, sirens, or megaphone announcements)?

Please select one option

SINGLE CODE

| Yes | 1 |  |
| --- | --- | --- |
| No | 2 |  |
| Prefer not to say | 96 |  |

CARER = 1-2 OR ‘CHILDREN’=1

Q7B. Does anyone you look after or care for have any other condition(s) that may stop them from hearing alarms or other noise alerts (such as a fire bell, sirens, or megaphone announcements)?

Please select one option

SINGLE CODE

| Yes | 1 |  |
| --- | --- | --- |
| No | 2 |  |
| Don’t know | 97 |  |
| Prefer not to say | 96 |  |

ASK ALL

Q8A. Is sign language your preferred language?

Please select one option

SINGLE CODE

| Yes | 1 |  |
| --- | --- | --- |
| No | 2 |  |
| Prefer not to say | 96 |  |

CARER = 1-2 OR ‘CHILDREN’=1

Q8B. Is sign language the preferred language of anyone you look after or care for?

Please select one option

SINGLE CODE

| Yes | 1 |  |
| --- | --- | --- |
| No | 2 |  |
| Don’t know | 97 |  |
| Prefer not to say | 96 |  |

ASK ALL

Q9A. Do you have a condition which can significantly affect your speech?

Please select one option

SINGLE CODE

| Yes | 1 |  |
| --- | --- | --- |
| No | 2 |  |
| Prefer not to say | 96 |  |

CARER = 1-2 OR ‘CHILDREN’=1

Q9B. Does anyone you look after or care for have a condition which can significantly affect their speech?

Please select one option

SINGLE CODE

| Yes | 1 |  |
| --- | --- | --- |
| No | 2 |  |
| Don’t know | 97 |  |
| Prefer not to say | 96 |  |

ASK ALL

Q10A. Are you blind or do you have significant difficulty seeing?

Please select one option

SINGLE CODE

| Yes | 1 |  |
| --- | --- | --- |
| Yes, but not when wearing contact lenses or glasses | 2 |  |
| No | 3 |  |
| Prefer not to say | 96 |  |

CARER = 1-2 OR ‘CHILDREN’=1

Q10B. Is anyone you look after or care for blind or do they have significant difficulty seeing?

Please select one option

SINGLE CODE

| Yes | 1 |  |
| --- | --- | --- |
| Yes, but not when wearing contact lenses or glasses | 2 |  |
| No | 3 |  |
| Don’t know | 97 |  |
| Prefer not to say | 96 |  |

ASK ALL

Q11A. Separate to a hearing or visual impairment, do you have a health condition that can make it hard to understand or talk to people at times?

Please select one option

SINGLE CODE

| Yes | 1 |  |
| --- | --- | --- |
| No | 2 |  |
| Prefer not to say | 96 |  |

CARER = 1-2 OR ‘CHILDREN’=1

Q11B. Separate to a hearing or visual impairment, does anyone you look after or care for have a health condition that can make it hard to understand or talk to people at times?

Please select one option

SINGLE CODE

| Yes | 1 |  |
| --- | --- | --- |
| No | 2 |  |
| Don’t know | 97 |  |
| Prefer not to say | 96 |  |

ASK ALL

Q12A. Do you currently have a reduced or impaired sense of smell which could prevent you from being able to sense a gas leak, burning / smoke, or soiled clothing / nappies?

Please select one option

SINGLE CODE

| Yes | 1 |  |
| --- | --- | --- |
| No | 2 |  |
| Prefer not to say | 96 |  |

CARER = 1-2 OR ‘CHILDREN’=1

Q12B. Does anyone you look after or care for currently have a reduced or impaired sense of smell which could prevent them from being able to sense a gas leak, burning / smoke, or soiled clothing / nappies?

Please select one option

SINGLE CODE

| Yes | 1 |  |
| --- | --- | --- |
| No | 2 |  |
| Don’t know | 97 |  |
| Prefer not to say | 96 |  |

ASK ALL

Q13A. Do you experience significant difficulty concentrating, remembering, or making decisions?

Please select one option

SINGLE CODE

| Yes | 1 |  |
| --- | --- | --- |
| No | 2 |  |
| Prefer not to say | 96 |  |

CARER = 1-2 OR ‘CHILDREN’=1

Q13B. Does anyone you look after or care for experience significant difficulty concentrating, remembering, or making decisions?

Please select one option

SINGLE CODE

| Yes | 1 |  |
| --- | --- | --- |
| No | 2 |  |
| Don’t know | 97 |  |
| Prefer not to say | 96 |  |

ASK ALL

Q14A. In an emergency, would you have significant difficulty doing the following?

Please select one option for each answer

SINGLE CODE

CAROUSEL, RANDOMISE STATEMENTS, FLIP SCALE

Answer Options

| Yes | 1 |  |
| --- | --- | --- |
| No | 2 |  |
| Prefer not to say | 96 | FIX |

Statements

| Hearing, understanding, or following spoken instructions in English on your own | 1 |  |
| --- | --- | --- |
| Speaking in English to someone you don’t know on your own | 2 |  |
| Moving about your home without aid or assistance | 3 |  |
| Dressing, bathing, or using the toilet on your own | 4 |  |
| Feeding yourself on your own | 5 |  |

CARER = 1-2 OR ‘CHILDREN’=1

Q14B. In an emergency, would anyone you look after or care for have significant difficulty doing the following?

Please select one option for each answer

SINGLE CODE

CAROUSEL, RANDOMISE STATEMENTS, FLIP SCALE

Answer Options

| Yes | 1 |  |
| --- | --- | --- |
| No | 2 |  |
| Don’t know | 97 | FIX |
| Prefer not to say | 96 | FIX |

Statements

| Hearing, understanding, or following spoken instructions in English on **their** own | 1 |  |
| --- | --- | --- |
| Speaking in English to someone **they** don’t know on **their** own | 2 |  |
| Moving about **their** home without aid or assistance | 3 |  |
| Dressing, bathing, or using the toilet on **their** own | 4 |  |
| Feeding themselves on **their** own | 5 |  |

ASK ALL

Q15A. Because of a disability and/or health condition, would you have significant difficulty doing errands **alone** such as visiting a pharmacy or shopping?

Please select one option

SINGLE CODE

| Yes | 1 |  |
| --- | --- | --- |
| No | 2 |  |
| Prefer not to say | 96 |  |

CARER = 1-2 OR ‘CHILDREN’=1

Q15B. Because of a disability and/or health condition, would anyone you look after or care for have significant difficulty doing errands **alone** such as visiting a pharmacy or shopping?

Please select one option

SINGLE CODE

| Yes | 1 |  |
| --- | --- | --- |
| No | 2 |  |
| Don’t know | 97 |  |
| Prefer not to say | 96 |  |

ASK ALL

Q16A. Do you have a condition or specific need (including for regular medication) that would be hard for you to explain to a doctor **on your own** without having it written down?

Please select one option

SINGLE CODE

| Yes | 1 |  |
| --- | --- | --- |
| No | 2 |  |
| Prefer not to say | 96 |  |

CARER = 1-2 OR ‘CHILDREN’=1

Q16B. Does anyone you look after or care for have a condition or specific need (including for regular medication) that would be hard for them to explain to a doctor **on their own** without having it written down?

Please select one option

SINGLE CODE

| Yes | 1 |  |
| --- | --- | --- |
| No | 2 |  |
| Don’t know | 97 |  |
| Prefer not to say | 96 |  |

ASK ALL

Q17. During an emergency, would your health or the health of anyone you care for (including infants) be significantly affected if you or they could not access or use any of the following:

Please select one option for each answer

SINGLE CODE

CAROUSEL, RANDOMISE STATEMENTS

Answer Options, FIX

| Yes – my health would be affected | 1 |  |
| --- | --- | --- |
| Yes – the health of someone I care for would be affected | 2 |  |
| Yes – **both** my health **and** the health of someone I care for would be affected | 3 |  |
| No – neither my health nor the health of someone I care for would be affected | 4 |  |
| Prefer not to say | 96 |  |

Statements

| A caregiver | 1 |  |
| --- | --- | --- |
| A service animal | 2 |  |
| Electrical devices that call for help if you fall, or have a problem at home (e.g. personal alarms, security systems including telecare) | 3 |  |
| Electrically powered assistive technology (e.g. communication aids/AAC) | 4 |  |
| Prescribed foods, feed, or feeding supplies | 5 |  |
| Other specific foods (e.g. due to sensory needs) that must be refrigerated or cooked | 6 |  |
| Medically prescribed food supplements | 7 |  |
| Refrigerated medications | 8 |  |
| Controlled medications / medications that are only prescribed for up to 30 days at a time | 9 |  |
| Other prescribed medications, including ointments and creams | 10 |  |
| Electric wheelchair / scooter | 11 |  |
| Home medical equipment that requires electricity (e.g. oxygen pumps, dialysis machines, CPAP devices, hoists, lifts) | 12 |  |
| Home medical equipment that does not rely on electricity (e.g. walkers, manual wheelchairs, specialist mattresses) | 13 |  |
| Equipment to help you manage your body temperature that is needed due to a medical condition | 14 |  |
| Sanitary products for medical conditions (e.g. urinary supplies like catheters and ostomy supplies) | 15 |  |
| Incontinence supplies (e.g. wearable pads) | 16 |  |
| Other medical supplies (e.g. syringes, blood sugar monitoring strips or oxygen cylinders) | 17 |  |

ASK ALL

Q18A. Apart from the equipment listed above, are there any other medical supplies or devices that may be difficult to access during a disaster and that are essential to your health?

Type your answer below

OPEN END

|  |
| --- |

CARER = 1-2 OR ‘CHILDREN’=1

Q18B. Apart from the equipment listed above, are there any other medical supplies or devices that may be difficult to access during a disaster and that are essential to the health of anyone you look after or care for?

Type your answer below

OPEN END

|  |
| --- |

# Part 2

## 2A Planning – Logistics

ASK ALL

Q19. Apart from emergency services, is there someone local you can contact for help or support in an emergency?

Please select one option

SINGLE CODE

| Yes | 1 |  |
| --- | --- | --- |
| No | 2 |  |
| Not sure | 97 |  |

ASK ALL

Q20. Do you have a plan for what to do if, because of a disaster, you had to leave your home in a hurry?

Please select one option

SINGLE CODE

| Yes | 1 |  |
| --- | --- | --- |
| No | 2 |  |
| Not sure | 97 |  |

ASK IF Q20 = 1 (Yes)

Q21. Would your plan to leave home quickly still work if there was no electricity or phone service?

Please select one option

SINGLE CODE

| Yes | 1 |  |
| --- | --- | --- |
| No | 2 |  |
| Not sure | 97 |  |

ASK ALL

Q22. Have you taken first aid training within the last 12 months?

Please select one option

SINGLE CODE

| Yes | 1 |  |
| --- | --- | --- |
| No | 2 |  |
| Not sure | 97 |  |

ASK ALL

Q23. Does your household have a fire escape plan?

Please select one option

SINGLE CODE

| Yes | 1 |  |
| --- | --- | --- |
| No | 2 |  |
| Not sure | 97 |  |

ASK ALL

Q24. Do you have an agreed meeting place for yourself and any loved ones in case you get separated and cannot return home?

Please select one option

SINGLE CODE

| Yes | 1 |  |
| --- | --- | --- |
| No | 2 |  |
| Not sure | 97 |  |

ASK ALL

Q25. Have you made a plan of what to take if you had to leave your home quickly?

Please select one option

SINGLE CODE

| Yes | 1 |  |
| --- | --- | --- |
| No | 2 |  |
| Not sure | 97 |  |

ASK ALL

Q26A. Have you prepared a ‘go-bag’ or bag of essentials that is always ready in case you or your household must leave home suddenly in a disaster?

Please select one option

SINGLE CODE

| Yes | 1 |  |
| --- | --- | --- |
| No | 2 |  |
| Not sure | 97 |  |

ASK IF CARER_HOUSEHOLD = 1-2 (Yes or Some)

Q26B. Do those you look after or care for **outside your household** have a ‘go-bag’ or bag of essentials prepared that is always ready in case they must leave home suddenly in a disaster?

Please select one option

SINGLE CODE

| Yes | 1 |  |
| --- | --- | --- |
| No | 2 |  |
| Not sure | 97 |  |

ASK ALL

Q27A. If you need to turn off the water, gas, and / or electricity supply to your home, do you know how to do this?

Please select one option

SINGLE CODE

| Yes | 1 |  |
| --- | --- | --- |
| Yes, but I would not be physically able to | 2 |  |
| No | 3 |  |
| Not sure | 97 |  |

ASK ALL

Q27B. Do you have family or friends that you and any loved ones you live with could stay with after a disaster, if you had to leave your neighbourhood?

Please select one option

SINGLE CODE

| Yes | 1 |  |
| --- | --- | --- |
| No | 2 |  |
| Not sure | 97 |  |

ASK ALL

Q27C. If you had to stay in a hotel / bed and breakfast for one week, would you have enough money to afford this?

Please select one option

SINGLE CODE

| Yes | 1 |  |
| --- | --- | --- |
| No | 2 |  |
| Not sure | 97 |  |
| Prefer not to say | 96 |  |

ASK IF EMP_STATUS = 1, 2, 3, 6, 11

Q28. If the electricity supply in your part of country suddenly failed, you might not be able to contact your school, university, workplace, or place where you volunteer. If this happened on a normal work- or school day morning, does your school, university, workplace, or place where you volunteer have an official plan that would tell you what to do?

Please select one option

SINGLE CODE

| Yes | 1 |  |
| --- | --- | --- |
| No | 2 |  |
| Don't know | 3 |  |

ASK IF Q28 = 1 (Yes)

Q29A. What would you do if this happened?

Please select one option

SINGLE CODE

| Follow the official plan by waiting at home for someone to contact me | 1 |  |
| --- | --- | --- |
| Follow the official plan by going to my local place of work or to my school / campus / university | 2 |  |
| Follow the plan by doing something else | 3 |  |
| Not follow the plan | 4 |  |
| I don’t know what I would do | 97 |  |

ASK IF Q28 = 2 (No)

Q29Bb. What would you do if this happened?

Please select one option

SINGLE CODE

| Wait at home for someone to contact me | 1 |  |
| --- | --- | --- |
| Go to my local place of work or to my school / campus / university | 2 |  |
| Follow an alternative plan | 3 |  |
| I don’t know what I would do | 97 |  |

ASK IF EMP_STATUS = 1, 2, 3, 6, 9, 10 or 11

Q30. If you couldn’t work or complete your normal responsibilities for one week and nobody could take over your tasks for you, do you believe it would have a significant impact on society and/or on other peoples’ wellbeing?

Please select one option

SINGLE CODE

| Yes | 1 |  |
| --- | --- | --- |
| Maybe | 2 |  |
| No | 3 |  |
| Don’t know | 97 |  |
| Prefer not to say | 96 |  |

## 2B Planning – Registration

ASK ALL

Q31. Have you heard about the new government mobile-alert system that sends you a message if there are potential dangers near you?

Please select one option

SINGLE CODE

| Yes | 1 |  |
| --- | --- | --- |
| Yes, and I received a message in the last six months | 2 |  |
| No | 3 |  |
| Not sure | 97 |  |

ASK ALL

Q32. Have you signed up for any other system that will contact you to tell you if there is a disaster in your area?

Please select one option

SINGLE CODE

| Yes | 1 |  |
| --- | --- | --- |
| No | 2 |  |
| Not sure | 97 |  |

ASK ALL

Q33. Are you or anyone **in your home** registered as a ‘vulnerable’ person with any of the following:

1. Your GP surgery or pharmacy
2. Your local council or local authority
3. Social care services
4. Your electricity, gas, water, or telephone company

Please select all that apply

MULTICODE

| Yes – I am registered | 1 |  |
| --- | --- | --- |
| Yes – Someone I care for is registered | 2 |  |
| I might be registered, but I’m not sure | 3 |  |
| Someone I care for might be registered, but I’m not sure | 4 |  |
| No | 5 |  |
| Prefer not to say | 96 | FIX |

ASK IF CARER_HOUSEHOLD = 1-2 (Yes or Some)

Q34. Is anyone you look after **outside of** your home registered as a ‘vulnerable’ person with any of the following:
CAROUSEL
1. GP surgery or pharmacy
2. Local council or local authority
3. Social care services
4. Electricity, gas, water, or telephone company
 5. Other (please specify)

Please select all that apply

MULTICODE

| Yes | 1 |  |
| --- | --- | --- |
| They might be registered, but I’m not sure | 2 |  |
| No | 3 |  |
| Prefer not to say | 96 | FIX |

ASK IF Q33 = 1 or 2

Q35. Are you or anyone you care for **in your home** registered as a ‘vulnerable’ person with any of the following:

Please select all that apply

MULTICODE, RANDOMISE

| Your GP surgery or pharmacy | 1 |  |
| --- | --- | --- |
| Your local council or local authority | 2 |  |
| Social care services | 3 |  |
| Your electricity, gas, water, or telephone company | 4 |  |
| Somewhere else (please specify) | 98 | FIX OE |
| Prefer not to say | 96 | FIX |

ASK IF Q34 = 1

Q36. Is anyone you look after **outside of** your home registered as a ‘vulnerable’ person with any of the following:

Please select all that apply

MULTICODE, RANDOMISE

| Their GP surgery or pharmacy | 1 |  |
| --- | --- | --- |
| Their local council or local authority | 2 |  |
| Social care services | 3 |  |
| Their electricity, gas, water, or telephone company | 4 |  |
| Somewhere else (please specify) | 98 | FIX OE |
| Prefer not to say | 96 | FIX |

# Prevalence of unmet needs

## 2C Written Information

ASK ALL

Q37. Do you have access to key documents, such as your birth certificate?

Please select one option

SINGLE CODE

| Yes | 1 |  |
| --- | --- | --- |
| No | 2 |  |
| Not sure | 97 |  |
| Prefer not to say | 96 |  |

INFO SCREEN:

In the following questions we will ask about information that you may have stored somewhere. This could be:

Written down on paper (e.g. in an address book)
Stored on your mobile phone (e.g. as a photo), but only if you have a spare battery pack for your phone
Stored online, or in an email to yourself

ASK ALL

Q38. Do you have copies of the following documents?

Please select one option for each answer

SINGLE CODE

CAROUSEL, RANDOMISE STATEMENTS, FLIP SCALE

Answer Options

| Yes | 1 |  |
| --- | --- | --- |
| No | 2 |  |
| Not sure | 97 | FIX |
| Not applicable | 99 | FIX |
| Prefer not to say | 96 | FIX |

Statements

| Important documents (e.g. passport, driver’s license, insurance policies). | 1 |  |
| --- | --- | --- |
| If your important documents do not match your gender or preferred name, a document that explains this | 2 |  |
| Contact information for loved ones | 3 |  |
| Contact information for key services (e.g. the power company, your local pharmacy) | 4 |  |
| Your doctor’s contact details | 5 |  |

ASK IF Q16A = 1

Q39A. You stated earlier that you have a condition or specific need that would be hard for you to explain to a doctor without having it written down. Do you have this information written down somewhere accessible?

Please select one option

SINGLE CODE

| Yes | 1 |  |
| --- | --- | --- |
| No | 2 |  |
| Not sure | 97 |  |

ASK IF Q16B = 1

Q39B. You said earlier that someone you care for or look after has a condition or specific need that would be hard for them to explain to a doctor without having it written down. Do they have this information written down somewhere accessible?

Please select one option

SINGLE CODE

| Yes | 1 |  |
| --- | --- | --- |
| No | 2 |  |
| Not sure | 97 |  |

ASK IF Q17_8, Q17_9, or Q17_10 = 1 OR 3

Q40A. Do you have copies of a written list of your medications, including how much you should take?

Please select one option

SINGLE CODE

| Yes | 1 |  |
| --- | --- | --- |
| No | 2 |  |
| Not sure | 97 |  |

ASK IF Q17_8, Q17_9, or Q17_10 = 2 OR-3

Q40B. Does the person you care for or look after have copies of a written list of their medications, including how much they should take?

Please select one option

SINGLE CODE

| Yes | 1 |  |
| --- | --- | --- |
| No | 2 |  |
| Not sure | 97 |  |

## 2D Smoke/CO Detectors

ASK ALL

Q41. Do you have a smoke detector on each floor of your home?

Please select one option

SINGLE CODE

| Yes | 1 |  |
| --- | --- | --- |
| No | 2 |  |
| Not sure | 97 |  |

ASK IF Q40 = 1 (Yes)

Q42. Has anyone checked your smoke detector(s) in the last six months?

Please select one option

SINGLE CODE

| Yes | 1 |  |
| --- | --- | --- |
| No | 2 |  |
| Not sure | 97 |  |

ASK ALL

Q43. Do you have a carbon monoxide detector on each floor of your home?

Please select one option

SINGLE CODE

| Yes | 1 |  |
| --- | --- | --- |
| No | 2 |  |
| Not sure | 97 |  |

ASK IF Q41 = 1 (Yes)

Q44. Has anyone checked your carbon monoxide detector in the last six months?

Please select one option

SINGLE CODE

| Yes | 1 |  |
| --- | --- | --- |
| No | 2 |  |
| Not sure | 97 |  |

## 2E Basic supplies

ASK ALL

Q45. If you had to leave your neighbourhood quickly today, and public transport was not available, would you and your household have sources of transport available that could transport both yourselves, and any baggage you would need to bring, to safety?

Please select one option

SINGLE CODE

| Yes | 1 |  |
| --- | --- | --- |
| No | 2 |  |
| Not sure | 97 |  |

ASK ALL

Q46. If there was no electricity or telephone service, would you have a way to receive information about disasters in your area other than word of mouth? (e.g. a solar-powered, hand-cranked or battery-operated radio, or a car radio)

Please select one option

SINGLE CODE

| Yes | 1 |  |
| --- | --- | --- |
| No | 2 |  |
| Not sure | 97 |  |

ASK ALL

Q47. If there was no electricity or gas supply to your home, your cooker and fridge would stop working, and you might not be able to buy food from the shops. If this were to happen, do you currently have enough food to feed everyone in your home (including babies) for a week?

Please select one option

SINGLE CODE

| Yes | 1 |  |
| --- | --- | --- |
| No | 2 |  |
| Not sure | 97 |  |

ASK ALL

Q48. Do you currently have enough personal hygiene supplies (e.g. soap, tampons, pads, loo roll, bin bags, hand sanitiser, moist wipes, etc.) for each member of your household for a week?

Please select one option

SINGLE CODE

| Yes | 1 |  |
| --- | --- | --- |
| No | 2 |  |
| Not sure | 97 |  |

ASK ALL

Q49. If it was winter and you had no heating, would you have enough sleeping bags or warm blankets for everyone in your household to stay warm?

Please select one option

SINGLE CODE

| Yes | 1 |  |
| --- | --- | --- |
| No | 2 |  |
| Not sure | 97 |  |

ASK ALL

Q50. In a power cut, cash machines and bank cards may stop working. Do you have enough cash to buy supplies?

Please select one option

SINGLE CODE

| Yes | 1 |  |
| --- | --- | --- |
| No | 2 |  |
| Not sure | 97 |  |
| Prefer not to say | 96 |  |

ASK ALL

Q51. Do you have any of the following items?

Please select one option for each answer

SINGLE CODE

CAROUSEL, RANDOMISE STATEMENTS, FLIP SCALE

Answer Options

| Yes | 1 |  |
| --- | --- | --- |
| No | 2 |  |
| Not sure | 97 | FIX |
| Not applicable | 99 | SHOW ONLY ON STATEMENT 7, FIX |

Statements

| A torch, headlamp, solar powered lantern, pack of glow sticks, or other battery-powered portable lighting? | 1 |  |
| --- | --- | --- |
| Extra batteries | 2 |  |
| Matches or lighters | 3 |  |
| A fire extinguisher | 4 |  |
| An extra battery pack for your phone | 5 |  |
| A spare phone charger | 6 |  |
| A spare set of home and car keys (if applicable) | 7 |  |
| A portable can opener (that you are able to use on your own and without electricity) | 8 |  |
| A first aid kit | 9 |  |

ASK ALL

Q52. If water from the taps was shut off, would you currently have at least 47 pints / 26.5 litres of non-tap water available per person in your home?

Please select one option

SINGLE CODE

| Yes | 1 |  |
| --- | --- | --- |
| No | 2 |  |
| Not sure | 97 |  |

ASK ALL

QC1. Select Green to progress

Please select one option

SINGLE CODE, RANDOMISE

| Blue | 1 | SCREEN OUT |
| --- | --- | --- |
| Green | 2 |  |
| Black | 3 | SCREEN OUT |
| Red | 4 | SCREEN OUT |
| Purple | 5 | SCREEN OUT |

## 2E Specialised supplies

ASK IF CHILDREN_AGE = 1 (0-2) AND THEY ENTER ‘1’ OR MORE AT CHILDREN_NUM

Q53. Do you have one week’s supply of disposable nappies?

Please select one option

SINGLE CODE

| Yes | 1 |  |
| --- | --- | --- |
| No | 2 |  |
| Not sure | 97 |  |

ASK IF Q5 = 1 (Yes)

Q54. If you, or a member of your household, are formula feeding an infant, do you/they have a way to do this without electricity? (e.g. for sterilising bottles, warming formula, etc.)

Please select one option

SINGLE CODE

| Yes | 1 |  |
| --- | --- | --- |
| No | 2 |  |
| Not sure | 97 |  |

ASK IF SCHOOL = 1 (Yes)

Q55. Does your child’s college, school, or nursery have an agreed meeting place in the event they need to evacuate their site?

Please select one option

SINGLE CODE

| Yes | 1 |  |
| --- | --- | --- |
| No | 2 |  |
| Not sure | 97 |  |

ASK IF PETS = 1 or more OR ANIMALS = 1 or more

Q56. If you own / are responsible for animals, do you have at least 1-week’s supply of food **and** spare non-tap water for each animal?

Please select one option

SINGLE CODE

| Yes | 1 |  |
| --- | --- | --- |
| No | 2 |  |
| Not sure | 97 |  |

ASK IF PETS = 1 or more OR ANIMALS = 1 or more

Q57. If the animal(s) you own / are responsible for take(s) medications, do you have a 2-week supply of extra medications for them?

Please select one option

SINGLE CODE

| Yes | 1 |  |
| --- | --- | --- |
| No | 2 |  |
| Not sure | 97 |  |
| Not applicable | 99 |  |

ASK IF PETS= 1 or more

Q58. If you had to evacuate your neighbourhood, would it be easy for you to take your pet(s) with you?

Please select one option

SINGLE CODE

| Very easy | 5 |  |
| --- | --- | --- |
| Easy | 4 |  |
| Difficult | 3 |  |
| Very difficult | 2 |  |
| Impossible | 1 |  |

ASK IF Q10A = 2 (Yes, but not when wearing contact lenses or glasses)

Q59A. Do you have extra glasses or contact lenses?

Please select one option

SINGLE CODE

| Yes | 1 |  |
| --- | --- | --- |
| No | 2 |  |
| This applies to me, but I don’t know the answer | 97 |  |
| This does not apply to me | 99 |  |
| Prefer not to say | 96 |  |

ASK IF Q10B = 2 (Yes, but not when wearing contact lenses or glasses)

Q59B. Does the person you care for or look after have extra glasses or contact lenses?

Please select one option

SINGLE CODE

| Yes | 1 |  |
| --- | --- | --- |
| No | 2 |  |
| This applies to them, but I don’t know the answer | 97 |  |
| This does not apply to them | 99 |  |
| Prefer not to say | 96 |  |

ASK IF Q6A = 1-2

Q60A. If you use hearing aids, do you have spare fully charged hearing aid batteries?

Please select one option

SINGLE CODE

| Yes | 1 |  |
| --- | --- | --- |
| No | 2 |  |
| This applies to me, but I don’t know the answer | 97 |  |
| This does not apply to me | 99 |  |
| Prefer not to say | 96 |  |

ASK IF Q6B = 1-2

Q60B. If anyone you care for uses hearing aids, do they have spare fully charged hearing aid batteries?

Please select one option

SINGLE CODE

| Yes | 1 |  |
| --- | --- | --- |
| No | 2 |  |
| This applies to them, but I don’t know the answer | 97 |  |
| This does not apply to them | 99 |  |
| Prefer not to say | 96 |  |

ASK ALL

Q61A. If you use dentures / false teeth, do you have spares?

Please select one option

SINGLE CODE

| Yes | 1 |  |
| --- | --- | --- |
| No | 2 |  |
| This applies to me, but I don’t know the answer | 97 |  |
| This does not apply to me | 99 |  |
| Prefer not to say | 96 |  |

ASK IF CARER = 1-2 OR CHILDREN = 1

Q61B. If anyone you care for uses dentures / false teeth, do they have spares?

Please select one option

SINGLE CODE

| Yes | 1 |  |
| --- | --- | --- |
| No | 2 |  |
| This applies to them, but I don’t know the answer | 97 |  |
| This does not apply to them | 99 |  |
| Prefer not to say | 96 |  |

ASK IF Q17_8, Q17_9 OR Q17_10 = 1 OR3

Q62A. If you take medications prescribed to you by your doctor, do you currently have two weeks’ worth of medications?

Please select one option

SINGLE CODE

| Yes | 1 |  |
| --- | --- | --- |
| No | 2 |  |
| This applies to me, but I don’t know the answer | 97 |  |
| This does not apply to me | 99 |  |
| Prefer not to say | 96 |  |

ASK IF Q17_8, Q17_9 OR Q17_10 = 2 OR 3

Q62B. If anyone you care for takes medications prescribed to them by their doctor, do they currently have two weeks’ worth of medications?

Please select one option

SINGLE CODE

| Yes | 1 |  |
| --- | --- | --- |
| No | 2 |  |
| This applies to them, but I don’t know the answer | 97 |  |
| This does not apply to them | 99 |  |
| Prefer not to say | 96 |  |

ASK IF Q17_5, Q17_6, Q17_7 OR Q17_17 = 1 OR 3

Q63A. Do you have 2-weeks’ worth of the following:

Please select one option for each answer

SINGLE CODE

CAROUSEL, RANDOMISE STATEMENTS

Answer Options

| Yes | 1 |  |
| --- | --- | --- |
| No | 2 |  |
| Don’t know | 97 |  |
| This does not apply to me | 99 |  |
| Prefer not to say | 96 |  |

Statements

| Prescribed foods, feed, or feeding supplies | 1 | PIPE IF Q17_5 = 1 OR 3 |
| --- | --- | --- |
| Other specific foods (e.g. due to sensory needs) that must be refrigerated or cooked | 2 | PIPE IF Q17_6 = 1 OR 3 |
| Medically prescribed food supplements | 3 | PIPE IF Q17_7 = 1 OR 3 |
| Other medical supplies (e.g. syringes, blood sugar monitoring strips or oxygen cylinders). | 4 | PIPE IF Q17_17 = 1 OR 3 |

ASK IF Q17_5, Q17_6, Q17_7 OR Q17_17 = 2 OR 3

Q63B. Do those you care for or look after have 2-weeks’ worth of the following:

Please select one option for each answer

SINGLE CODE

CAROUSEL, RANDOMISE STATEMENTS

Answer Options

| Yes | 1 |  |
| --- | --- | --- |
| No | 2 |  |
| This applies to them, but I don’t know the answer | 97 |  |
| This does not apply to them | 99 |  |
| Prefer not to say | 96 |  |

Statements

| Prescribed foods, feed, or feeding supplies | 1 | PIPE IF Q17_5 = 2 OR 3 |
| --- | --- | --- |
| Other specific foods (e.g. due to sensory needs) that must be refrigerated or cooked | 2 | PIPE IF Q17_6 = 2 OR 3 |
| Medically prescribed food supplements | 3 | PIPE IF Q17_7 = 2 OR 3 |
| Other medical supplies (e.g. syringes, blood sugar monitoring strips or oxygen cylinders). | 4 | PIPE IF Q17_17 = 2 OR 3 |

ASK IF Q17_8, Q17_11, OR Q17_12 = 1 OR3

Q64A. Do you have a plan for an alternate power source for medical equipment or refrigerated medication in the event of a power cut?

Please select one option

SINGLE CODE

| Yes | 1 |  |
| --- | --- | --- |
| No | 2 |  |
| This applies to me, but I don’t know the answer | 97 |  |
| This does not apply to me | 99 |  |
| Prefer not to say | 96 |  |

ASK IF Q17_8, Q17_11, OR Q17_12 = 2 OR 3

Q64B. Do those you care for or look after have a plan for an alternate power source for medical equipment or refrigerated medication in the event of a power cut?

Please select one option

SINGLE CODE

| Yes | 1 |  |
| --- | --- | --- |
| No | 2 |  |
| This applies to them, but I don’t know the answer | 97 |  |
| This does not apply to them | 99 |  |
| Prefer not to say | 96 |  |

ASK IF Q17_8 = 1 OR3

Q65A. Do you have a small cooler, portable cool box or bag, and / or cold packs or freezer bricks for keeping refrigerated medications cold if you evacuate?

Please select one option

SINGLE CODE

| Yes | 1 |  |
| --- | --- | --- |
| No | 2 |  |
| This applies to me, but I don’t know the answer | 97 |  |
| This does not apply to me | 99 |  |
| Prefer not to say | 96 |  |

ASK IF Q17_8 = 2 OR 3

Q65B. Do those you look after have a small cooler, portable cool box or bag, and / or cold packs or freezer bricks for keeping refrigerated medications cold if they evacuate?

Please select one option

SINGLE CODE

| Yes | 1 |  |
| --- | --- | --- |
| No | 2 |  |
| This applies to them, but I don’t know the answer | 97 |  |
| This does not apply to them | 99 |  |
| Prefer not to say | 96 |  |

ASK IF Q17_14 = 1 OR 3

Q66A. Do you have equipment to manage your temperature that will work in a power cut? E.g. heat pads, battery operated fans, etc.

Please select one option

SINGLE CODE

| Yes | 1 |  |
| --- | --- | --- |
| No | 2 |  |
| This applies to me, but I don’t know the answer | 97 |  |
| This does not apply to me | 99 |  |
| Prefer not to say | 96 |  |

ASK IF Q17_14 = 2 OR 3

Q66B. Do those you care for or look after have equipment to manage their temperature that will work in a power cut? E.g. heat pads, battery operated fans, etc.

Please select one option

SINGLE CODE

| Yes | 1 |  |
| --- | --- | --- |
| No | 2 |  |
| This applies to them, but I don’t know the answer | 97 |  |
| This does not apply to them | 99 |  |
| Prefer not to say | 96 |  |

ASK IF Q17_16 = 1 OR3

Q67A. Do you have incontinence supplies?

Please select one option

SINGLE CODE

| Yes | 1 |  |
| --- | --- | --- |
| No | 2 |  |
| This applies to me, but I don’t know the answer | 97 |  |
| This does not apply to me | 99 |  |
| Prefer not to say | 96 |  |

ASK IF Q17_16 = 2 OR 3

Q67B. Do those you care for or look after have incontinence supplies?

Please select one option

SINGLE CODE

| Yes | 1 |  |
| --- | --- | --- |
| No | 2 |  |
| This applies to them, but I don’t know the answer | 97 |  |
| This does not apply to them | 99 |  |
| Prefer not to say | 96 |  |

# Perceptions #2

ASK ALL

Q68. How prepared would you say you were for the practicalities of the COVID-19 pandemic?

Please select one option

SINGLE CODE

| Very prepared | 3 |  |
| --- | --- | --- |
| Somewhat prepared | 2 |  |
| Not prepared | 1 |  |

ASK ALL

Q69. Did you experience practical difficulties because of the COVID-19 pandemic?

Please select one option

SINGLE CODE

| Yes – significant practical difficulties | 1 |  |
| --- | --- | --- |
| Some difficulties | 2 |  |
| No difficulties | 3 |  |
| Prefer not to say | 96 |  |

ASK ALL

Q70. Have you previously experienced a power cut lasting 24 hours or more?

Please select one option

SINGLE CODE

| Yes, in the last five years | 1 |  |
| --- | --- | --- |
| Yes, but more than five years ago | 2 |  |
| No | 3 |  |
| Not sure | 97 |  |

ASK ALL

Q71. Do you personally know anyone (excluding yourself) who has experienced a power cut lasting 24 hours or more?

Please select one option

SINGLE CODE

| Yes, in the last five years | 1 |  |
| --- | --- | --- |
| Yes, but more than five years ago | 2 |  |
| No | 3 |  |
| Not sure | 97 |  |

Click or tap here to enter text.

ASK ALL

Q72. Apart from the COVID-19 pandemic, have you experienced any other kind of disaster before?

Please select one option

SINGLE CODE

| Yes | 1 |  |
| --- | --- | --- |
| No | 2 |  |
| Not sure | 97 |  |
| Prefer not to say | 96 |  |

Click or tap here to enter text.

ASK ALL

Q73. How likely do you think a widespread power cut lasting two or more days is?

Please select one option

SINGLE CODE

| Very likely | 4 |  |
| --- | --- | --- |
| Likely | 3 |  |
| Unlikely | 2 |  |
| Very unlikely | 1 |  |
| Don’t know | 97 |  |

ASK ALL

Q74. To what extent do you think power outage / power cuts pose a risk to…

Please select one option for each answer

SINGLE CODE

CAROUSEL, RANDOMISE STATEMENTS, FLIP SCALE

Answer Options

| Major risk | 5 |  |
| --- | --- | --- |
| Significant risk | 4 |  |
| Moderate risk | 3 |  |
| Minor risk | 2 |  |
| No risk at all | 1 |  |
| Don't know | 97 |  |

Statements

| People in the UK | 1 |  |
| --- | --- | --- |
| You personally | 2 |  |
| Please select ‘no risk at all’ | 3 | IF **DO NOT** SELECT OPTION 1, SCREEN OUT |

ASK ALL

Q75. How confident do you feel that you/your household can cope with a power cut of two days?

Please select one option

SINGLE CODE

| Very confident | 4 |  |
| --- | --- | --- |
| Confident | 3 |  |
| Unconfident | 2 |  |
| Very unconfident | 1 |  |
| Don’t know | 97 |  |
| Prefer not to say | 96 |  |

ASK ALL

Q76. Is there something that would make you feel more prepared to cope with a power cut of 2 days or more?

Type your answer below

OPEN END

|  |
| --- |

ASK ALL

Q77. In some countries, the government runs drills or exercises to help people practice what they would do in a disaster. If the UK government decided to run an exercise like this that would take one hour of your time, would you be interested in taking part?

Please select one option

SINGLE CODE

| Yes, definitely | 5 |  |
| --- | --- | --- |
| Probably | 4 |  |
| Maybe | 3 |  |
| Probably not | 2 |  |
| Definitely not | 1 |  |
| Don’t know | 97 |  |

ASK ALL

Q78. Other than information about the COVID-19 pandemic, have you looked for information on preparing for disasters in the past?

Please select one option

SINGLE CODE

| Yes | 1 |  |
| --- | --- | --- |
| No | 2 |  |
| Don’t know | 97 |  |

# Self description # 2

ASK ALL

QC2. Which of the following best describes your current working status?

Please select one option that best describes the majority of your time

SINGLE CODE

| Working full time - working 30 hours per week or more – or on leave from a full-time job. | 1 |  |
| --- | --- | --- |
| Working part time – guaranteed hours, working up to 29 hours per week, or on leave from a part-time job. | 2 |  |
| Working part time – zero hours contract | 3 |  |
| Not working but seeking work | 4 |  |
| Not working and not seeking work, **due to** long-term illness or disability | 5 |  |
| Not working and not seeking work, for reasons **other than** long term illness or disability |  |  |
| Student | 6 |  |
| Retired on a state pension only | 7 |  |
| Retired with a private pension | 8 |  |
| Stay-at-home spouse/partner | 9 |  |
| Unpaid carer | 10 |  |
| Unpaid volunteer | 11 |  |
| Not listed (please specify) | 98 | FIX OE |
| Prefer not to say | 96 | FIX |

ASK ALL

Q79. Would you consider yourself to be ‘vulnerable’ during a disaster?

We asked you this question at the start of the survey, but we are interested to know whether thinking about the questions in this survey has made you change your mind.

Please select one option

SINGLE CODE

| Yes | 1 |  |
| --- | --- | --- |
| Maybe | 2 |  |
| No | 3 |  |
| Don’t know | 97 |  |
| Prefer not to say | 96 |  |

ASK ALL

Q80A. Would you consider anyone else in your household to be ‘vulnerable’ during a disaster?

We asked you this question at the start of the survey, but we are interested to know whether thinking about the questions in this survey has made you change your mind.

Please select one option

SINGLE CODE

| Yes | 1 |  |
| --- | --- | --- |
| Maybe | 2 |  |
| No | 3 |  |
| Don’t know | 97 |  |
| Prefer not to say | 96 |  |

ASK IF CARER_HOUSEHOLD = 1-2 (Yes or Some)

Q80B. Would you consider anyone you look after or care for **outside** your household for to be vulnerable during a disaster?

We asked you this question at the start of the survey, but we are interested to know whether thinking about the questions in this survey has made you change your mind.

Please select one option

SINGLE CODE

| Yes | 1 |  |
| --- | --- | --- |
| Maybe | 2 |  |
| No | 3 |  |
| Don’t know | 97 |  |
| Prefer not to say | 96 |  |

ASK ALL

Q81. Would you be interested in learning more about preparing for disasters in the future?

Please select one option

SINGLE CODE

| Yes definitely | 5 |  |
| --- | --- | --- |
| Probably | 4 |  |
| Maybe | 3 |  |
| Probably not | 2 |  |
| Definitely not | 1 |  |
| Don’t know | 97 |  |

ASK ALL

Q82. What format would you want information on preparedness to take?

Please select all that apply

MULTICODE, RANDOMISE

| Video | 1 |  |
| --- | --- | --- |
| Written leaflet | 2 |  |
| Posters in community hubs | 3 |  |
| Mobile app | 4 |  |
| Webpage |  |  |
| Not listed (please specify) | 98 | FIX OE |
| Don't know | 97 | FIX EXCLUSIVE |

# Closing Screen:

Thank you for completing this survey!

The purpose of this survey is to provide data that can help support work to prepare the UK for a range of events described in the National Risk Register, including events that have occurred around the UK recently such as storms, floods and power outages. For more information about the national risk register, please visit: <https://www.gov.uk/government/publications/national-risk-register-2020>

In case you would like more information about preparing for disasters, below are some useful websites with information on emergency preparedness:

- UK Government information on preparing for emergencies: <https://www.gov.uk/government/publications/preparing-for-emergencies/preparing-for-emergencies>
- A leaflet on making a Household Emergency Plan: <https://www.westsussex.gov.uk/media/9340/household_emergency_plan.pdf>
- Information about preparing for an emergency: <https://cheshireresilience.org.uk/how-to-prepare-for-an-emergency/>
- Red Cross website about preparing an emergency kit for your home, your car, and for on the move: <https://www.redcross.org.uk/get-help/prepare-for-emergencies/prepare-an-emergency-kit>
- Disaster preparedness advice specifically for trans and non-binary people: <https://transequality.org/issues/resources/hurricane-preparedness-info-trans-people>
- Diabetes care during emergencies: <https://www.cdc.gov/diabetes/library/features/diabetes-care-during-emergencies.html?CDC_AA_refVal=https%3A%2F%2Fwww.cdc.gov%2Fdiabetes%2Fmanaging%2Fpreparedness.html>
- A guide for making arrangements for pets, livestock and larger animals during an emergency: <https://services.swale.gov.uk/assets/Major-Emergencies/A-guide-for-making-arrangements-for-pets-larger-animals-and-livestock-during-an-emergency-WEB%20A%20(002)xx.pdf>
- And finally, remember to check your smoke and carbon monoxide alarms using this reminder tool: <https://www.safelincs.co.uk/reminders/>.

**Acknowledgement**

This survey is based on the Household Emergency Preparedness Instrument created for US-based audiences by Heagale and colleagues (2020). It has been revised with expert input from the NIHR Health Protection Research Unit in Emergency Preparedness and Response for use in the UK context.

**References:**

Heagele, T. N., McNeill, C. C., Adams, L. M., & Alfred, D. M. (2020). Household Emergency Preparedness Instrument Development: A Delphi Study. *Disaster Medicine and Public Health Preparedness*, 1–13. https://doi.org/10.1017/dmp.2020.292

If you have any further comments or feedback, please use the box below, and thank you again for completing this survey.

| <Please type here> |
| --- |
